# Long COVID shows IL-1 pathway dysregulation without a discrete circulating biomarker signature

**DOI:** 10.3389/fimmu.2026.1957339

**Published:** 2026-10-19

**Authors:** Francisco Muñoz-López, Marissa Flores-Santamaría, Rebeca Santano, Macedonia Trigueros, Eva Riveira-Muñoz, Sara Cabrero de las Heras, Gooya Banaei, Edwards Pradenas, Benjamin Trinité, Silvia Marfil, Ana Gervassi, Víctor Urrea, Jon Izquierdo-Pujol, Cora Loste, José Ramón Santos, Gemma Lladós, Tomasso Francesco Aiello, Anna Chamorro, Alfons Jiménez, Carlota Dobaño, Roger Paredes, Julià Blanco, Ester Ballana, Gemma Moncunill, Lourdes Mateu, Maria Nevot, Marta Massanella

**Affiliations:** 1 IrsiCaixa, Badalona, Barcelona, Spain; 2 ISGlobal, Barcelona, Spain; 3 Faculty of Medicine and Health Sciences, Universitat de Barcelona (UB), Barcelona, Spain; 4 Centro de Investigación Biomédica en Red de Enfermedades Infecciosas (CIBERINFEC), Instituto de Salud Carlos III (ISCIII), Madrid, Spain; 5 Germans Trias i Pujol Research Institute (IGTP), Badalona, Spain; 6 Facultad de Ciencias, Universidad San Sebastián, Concepción, Chile; 7 Biomedicine Doctoral Programme, Faculty of Biology, University of Barcelona (UB), Barcelona, Spain; 8 Infectious Diseases Department, Germans Trias i Pujol Hospital, Badalona, Spain; 9 Fight Against Infections Foundation, Badalona, Spain; 10 Chair in Infectious Diseases and Immunity, Centre for Health and Social Care Research (CESS), Faculty of Medicine, University of Vic-Central University of Catalonia (UVic-UCC), Vic, Spain; 11 Spanish Long COVID Research Network (REiCOP), Madrid, Spain; 12 Centro de Investigación Biomédica en Red de Epidemiología y Salud Pública (CIBERESP), Barcelona, Spain; 13 Center for Global Health and Diseases, Department of Pathology, Case Western Reserve University School of Medicine, Cleveland, OH, United States; 14 Department of Infectious Diseases and Immunity, University of Vic-Central University of Catalonia (UVic-UCC), Vic, Spain; 15 CaixaResearch Institute, “la Caixa” Foundation, Barcelona, Spain

**Keywords:** EBV reactivation-associated serology, IL-1ra, immune dysregulation, PASC, plasma SARS-CoV-2 RNA, vascular biomarkers

## Abstract

**Introduction:**

Long COVID (LC) is characterized by persistent and, often, disabling symptoms, yet its biological basis remains unclear. Multiple mechanisms, including immune dysregulation, endothelial dysfunction, and persistence of SARS-CoV-2 components, have been proposed, although it is unknown whether these define distinct biological subtypes or overlapping processes.

**Methods:**

We conducted a cross-sectional analysis of 312 individuals, including 139 people with LC (PWLC), 99 COVID-19-recovered controls (both groups sampled a median of 8 months after primary SARS-CoV-2 infection), and 74 uninfected controls, from an unvaccinated population, thereby minimizing major sources of biological and epidemiological variability. Circulating immune and endothelial biomarkers and plasma SARS-CoV-2 RNA were assessed, together with serological signatures associated with herpesvirus reactivation and SARS-CoV-2-specific humoral responses. Associations with symptom persistence were analyzed using multivariable models and machine learning approaches.

**Results:**

Most soluble biomarker differences primarily distinguished previously infected individuals from uninfected controls rather than PWLC from recovered participants. Increased IL-1 receptor antagonist (IL-1RA) levels, together with reduced IL-1β/IL-1RA ratios, were the immune alterations most strongly associated with LC, while P-selectin was the endothelial marker showing a suggestive association with symptom persistence. Plasma SARS-CoV-2 RNA was detected at low and comparable frequencies in PWLC and recovered individuals, while Ct values tended to be lower among RNA-positive PWLC not reaching statistical difference. Epstein-Barr virus serological profiles were characterized by lower latency- and higher reactivation-associated responses in PWLC, whereas SARS-CoV-2-specific antibody levels were comparable between groups. Integrated biomarker profiles discriminated prior infection more effectively than LC status, and expansion to multimodal predictors did not clearly improve discrimination between PWLC and recovered participants.

**Discussion:**

Overall, LC was associated with overlapping immune, endothelial, and virological alterations rather than discrete biomarker-defined subtypes.

## 1 Introduction

Long COVID (LC) is characterized by persistent or relapsing symptoms following SARS-CoV-2 infection, even in individuals with mild acute COVID-19 (1). These manifestations span multiple organ systems, including the cardiovascular, nervous, endocrine, gastrointestinal, reproductive, and immune systems, and are frequently associated with impaired daily functioning and reduced quality of life (2) Meta-analyses estimate that up to 30-40% of people infected report ongoing symptoms in pre-vaccination cohorts (3), whereas lower estimates (around 6-7%) have been described in more recent, largely post-vaccination populations using stricter clinical definitions (4, 5). LC contributes to loss of productivity and long-term physical, social, and psychological disability, representing an increasing public health burden as well as a deep individual impact (2) This complexity highlights the need for mechanistic studies capable of identifying biological phenotypes and biomarkers to support people with LC (PWLC) stratification and targeted interventions.

Current pathophysiological models propose a multifactorial process involving partially overlapping and non-mutually exclusive mechanisms, including immune dysregulation, endothelial damage, viral persistence and reactivation of latent viruses (6). Sustained alterations in cytokine and chemokine profiles and immune cell function, particularly involving T-cell activation and exhaustion, have been reported in some cohorts (7–10). In parallel, endothelial activation, platelet activation, and microvascular dysfunction have been implicated in LC, with studies reporting increased levels of vascular and pro-thrombotic biomarkers as well as fibrin(ogen) amyloid microclots, which could impair tissue perfusion and thereby explain some of the common symptoms such as fatigue, dyspnea and cognitive impairment (11–13). In addition, detection of SARS-CoV-2 RNA or protein in diverse tissues months after infection further suggests a role for ongoing antigenic stimulation as a driver of inflammation and symptomatology (14, 15). This may interact with reactivation of latent herpesviruses, particularly Epstein-Barr virus (EBV), reported in LC cohorts and linked to persistent immune perturbations (16). However, these findings have not been consistently reproduced across studies, and their relationship to persistent symptoms remains unclear.

A major limitation of the current literature is the heterogeneity in study design and case definition, which hampers robust comparisons and meta-analytic synthesis. Many cohorts differ in timing of sampling after infection, dominant viral variants, background immunity and exposure, and frequently combined vaccinated and unvaccinated individuals, as well as participants with one or multiple infections (4). In this context, unraveling the effects of host factors from those of prior vaccination, hybrid immunity or repeated antigenic exposure remains challenging, and may partly explain the lack of consistency in reported immune and endothelial signatures of LC. There is therefore an urgent need for well-characterized, homogeneous cohorts that minimize sources of biological and epidemiological variability. In particular, the study of unvaccinated individuals following primary SARS-CoV-2 infection and without known reinfection offers a useful framework to identify immune and endothelial alterations specifically associated with LC, without the confounding influence of vaccine-induced immunity or repeated exposure.

Here, we investigated immune and endothelial biomarkers, circulating SARS-CoV-2 RNA, SARS-CoV-2-specific humoral immunity, and reactivation-associated herpesvirus serological profiles in a well-characterized cohort of unvaccinated individuals after laboratory-confirmed or clinically diagnosed primary SARS-CoV-2 infection. By integrating multiple biological domains within a homogeneous population, we aimed to determine whether LC is associated with discrete biomarker-defined subtypes or with convergent dysregulation across pathways.

## 2 Materials and methods

### 2.1 Study design and participants

The LoNK-COVID study (PI-23-035) was a cross-sectional observational study conducted at the Germans Trias i Pujol Hospital. Participants were primarily recruited from the KING cohort extension study (PI-20-217), a dynamic cohort established during the acute phase of the pandemic. KING initially included individuals followed after acute COVID-19, including hospitalized patients, and was subsequently expanded to include referrals to the Long COVID Unit. For the present study, all available KING participants who met the predefined eligibility criteria and had sufficient stored plasma were included. Eligible participants were unvaccinated at the time of sampling, and PWLC and recovered had a laboratory-confirmed or clinically diagnosed primary SARS-CoV-2 infection 5–14 months before sample collection, and had no documented or clinically suspected reinfection before sampling. Potential reinfections were assessed through review of available medical records, participant interviews/questionnaires regarding new compatible acute episodes, and documented PCR or antigen test results; when longitudinal serological samples were available, changes in SARS-CoV-2 antibody levels were also considered. PWLC and recovered participants were not individually matched.

A total of 312 participants were included and classified into three groups: PWLC (n=139), COVID-19 recovered individuals without persistent symptoms (Recovered, n=99), and SARS-CoV-2 uninfected controls (Uninfected, n=74). The KING cohort contributed all 139 PWLC, 99 recovered participants, and 55 contemporaneous uninfected controls. KING uninfected controls had no history or symptoms suggestive of SARS-CoV-2 infection and had negative PCR and/or serological testing. An additional 19 pre-pandemic uninfected controls were included from the XMRV and APOPV^+^I^−^ studies, in which they had participated as uninfected controls. These samples were selected based on availability of sufficient stored plasma and, by virtue of their pre-pandemic collection, had no previous SARS-CoV-2 exposure (**Figure 1**). Blood samples were collected in EDTA tubes, and plasma was separated by centrifugation, aliquoted and stored at -80°C until analysis.

**Figure 1:**
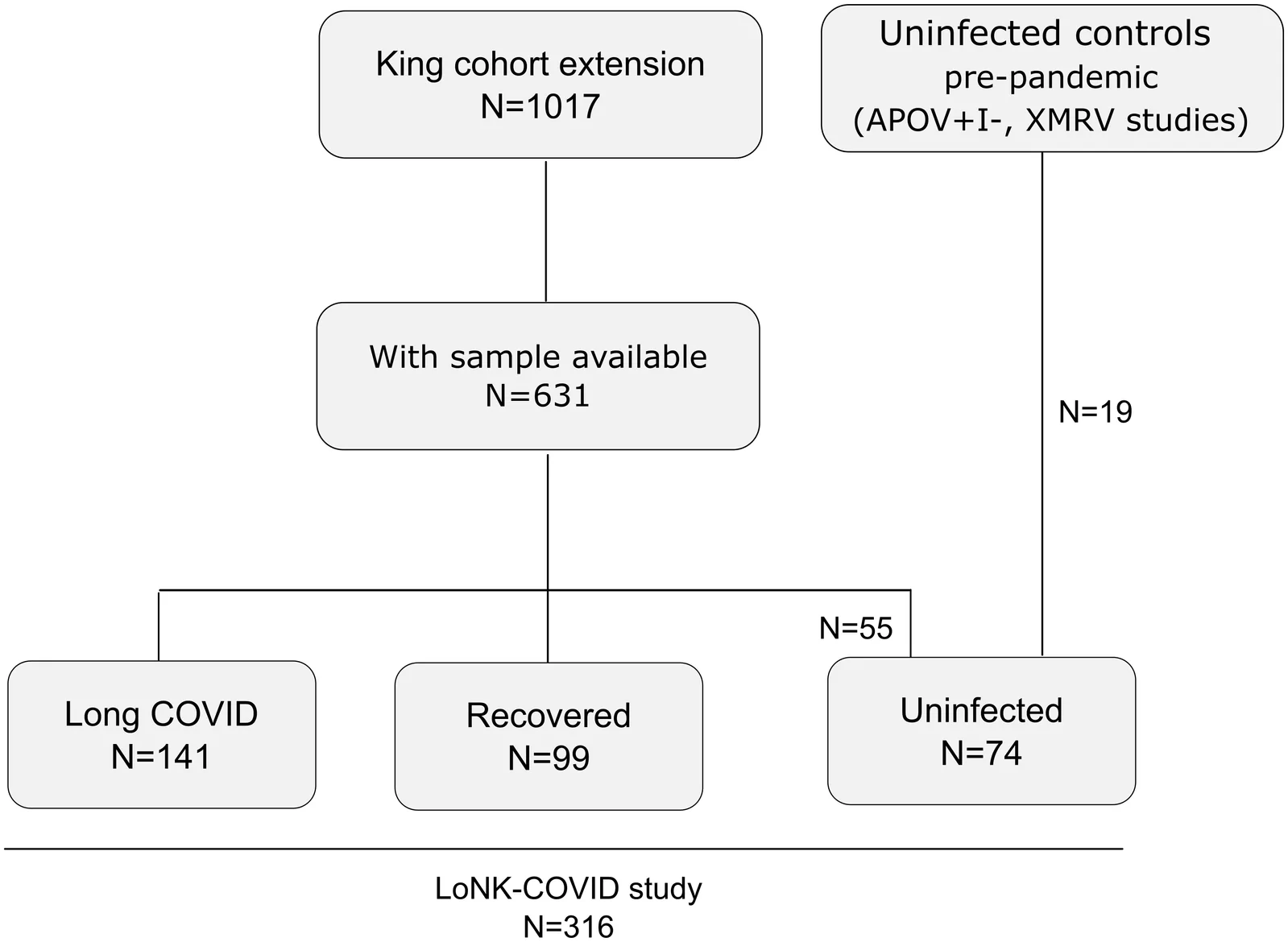
Cohort design flowchart.

The diagnosis of LC followed WHO criteria and was established by a physician from the Long COVID Unit. PWLC were required to present persistent or relapsing SARS-CoV-2-related symptoms lasting ≥3 months after acute infection that could not be explained by an alternative diagnosis. LC diagnosis was based on specialist clinical assessment, including medical history, physical examination, and routine laboratory and diagnostic investigations as clinically indicated to exclude alternative causes of persistent symptoms. Symptoms and functional assessments were systematically collected during face-to-face visits using prespecified questionnaires. Symptoms were considered persistent when present at the time of study assessment and reported as having developed after, persisted from, or worsened following the acute episode. Acute infection was confirmed by PCR, rapid antigen test, or serology when available. For some PWLC infected during the first epidemic wave, when access to diagnostic testing was limited, laboratory confirmation of the acute infection was unavailable and a clinical diagnosis of COVID-19 was accepted based on a compatible clinical presentation and/or close contact with a confirmed SARS-CoV-2 case. The complete list of symptoms assessed to support LC diagnosis and exclude alternative conditions are provided in **Supplementary Table 1**.

Written informed consent for sample collection and future research use was obtained at enrolment under the original cohort protocols (KING Cohort extension, APOPV^+^I^-^, and XMRV studies), conducted in accordance with Good Clinical Practice and GDPR 2016/679. The LoNK-COVID protocol was approved by the Germans Trias i Pujol Hospital Ethics Committee (HUGTiP/PI-23-035) and authorized the secondary use of these samples and associated clinical data.

### 2.2 Measurement of immune and endothelial biomarkers

Cytokine and chemokine levels were measured on plasma samples using the 30-Plex Panel for Luminex (Life Technologies, LHC6003), including: granulocyte colony-stimulating factor (G-CSF), epidermal growth factor (EGF), granulocyte-macrophage colony-stimulating factor (GM-CSF), fibroblast growth factor 2 (FGF-basic), interferon alpha (IFN-α), monocyte chemoattractant protein 1 (MCP-1), hepatocyte growth factor (HGF), interferon gamma (IFN-γ), monokine induced by interferon gamma (MIG), vascular endothelial growth factor (VEGF), interferon gamma-induced protein 10 (IP-10), interleukin 1 beta (IL-1β), macrophage inflammatory protein 1 alpha (MIP-1α), interleukin 1 receptor antagonist (IL-1RA), macrophage inflammatory protein 1 beta (MIP-1β), interleukin 2 (IL-2), chemokine (C-C motif) ligand 5 (regulated on activation, normal T-cell expressed and secreted, RANTES), interleukin 2 receptor (IL-2R), interleukin 4 (IL-4), interleukin 5 (IL-5), interleukin 6 (IL-6), interleukin 7 (IL-7), interleukin 8 (IL-8), interleukin 10 (IL-10), interleukin 12 (IL-12; p40/p70), interleukin 13 (IL-13), interleukin 15 (IL-15), interleukin 17 (IL-17), tumor necrosis factor alpha (TNF-α), and eotaxin, following manufacturer’s instructions.

Plasma concentrations of angiopoietin-1 (ANG-1, EHANGPT1), angiopoietin-2 (ANG-2, KHC1641), and soluble P-selectin (BMS219-4) from Life Technologies, and soluble endothelial protein C receptor (sEPCR, DY2245) and endothelin-1 (ET-1, DET100) from R&D Systems were measured as endothelial damage markers by ELISA according to the manufacturers’ instructions. We also quantified the levels of transforming growth factor beta (TGF-β) by ELISA (Life Technologies, BMS249-4) according to the supplier’s protocol.

For both Luminex and ELISAs, samples from the different study groups were distributed across assay plates to minimize potential batch effects.

### 2.3 SARS-CoV-2 RNA detection

To detect SARS-CoV-2 RNA in plasma, a two-step nested RT-qPCR targeting the N and E genes was performed in participants with sufficient plasma available (1 mL), including 139 PWLC, 94 recovered individuals, and 11 pre-pandemic uninfected controls. 1 mL of plasma was centrifuged at 25,500 x g for 60 minutes at 4°C. The pellet was resuspended in approximately 200 µL of the remaining supernatant. Viral RNA was extracted using the Maxwell HT Viral TNA kit (Promega) on a KingFisher instrument and eluted in 40 µL of nuclease-free water. Viral detection was performed using a two-step nested qPCR protocol to increase sensitivity. In the first amplification step, two independent regions of the SARS-CoV-2 genome were targeted (N and E genes) with specific primer sets (Cov2N2 F/R and NestedStarsE 3Fwd/Rev, 2 wells per gene) with parallel amplification of the human RNase P gene as an internal control (RNaseP Forward/Reverse, 1 well). Reverse transcription was performed at 50°C for 15 minutes, followed by 22 cycles of 95°C for 3 seconds and 55°C for 30 seconds, using 5 µL of input RNA. For the second amplification step, the entire first-round amplification product was distributed across multiple real-time qPCR reactions targeting the N and E genes. Specifically, 5 µL of the first-round PCR product were transferred into each of four independent second-round reactions for the N target and four independent reactions for the E target, thereby maximizing the use of the amplified product and increasing the sensitivity of viral RNA detection. In parallel, the human RNase P control was analyzed in quadruplicate for each first-round amplification. Primers and probes were obtained from the CDC 2019-nCoV Real-Time RT-PCR Diagnostic Panel and the E Sarbeco assay (Integrated DNA Technologies). Amplification was performed for 45 cycles at 95°C for 3 seconds and 55°C for 30 seconds on an Applied Biosystems 7500 or QuantStudio 5 Real-Time PCR system (Thermo Fisher Scientific). A sample was considered positive if at least one replicate for either the N or E target showed a valid amplification curve within the 45-cycle run; no additional numerical cycle threshold (Ct) cutoff was prespecified. The RT-qPCR component was based on previously established methodology using the CDC N-target primer/probe system (17), with a nested pre-amplification step introduced in the present study to increase sensitivity for low-abundance plasma SARS-CoV-2 RNA. A formal analytical limit of detection was not established for the complete nested workflow. Low-level positive samples were not systematically confirmed by repeat testing of the original RNA extract or by independent RNA re-extraction. Each experimental run included negative extraction controls, no-template controls, and a SARS-CoV-2-positive control. Negative extraction controls were processed in parallel with study samples through RNA extraction and amplification, and no-template controls were included during amplification; all remained negative throughout the experiments. The positive control was used at a low concentration producing low-level amplification to minimize the amount of viral template introduced into the workflow and reduce the risk of carryover contamination. Pre- and post-amplification procedures were performed in physically separated areas.

### 2.4 Humoral responses against SARS-CoV-2 and other viruses

Plasma samples (dilution 1/500) were analyzed using a high-throughput multiplex quantitative suspension array (Luminex) to measure IgG, IgM, and IgA antibodies against SARS-CoV-2 and other viral antigens, as previously described (18). For IgM and IgA assays, samples were pretreated with 1:10 anti-Human IgG (GullSORB™), to deplete IgG and avoid its interference. The multiplex panel included five SARS-CoV-2 antigens: full-length nucleocapsid (N FL), full-length spike (S), spike subunits 1 and 2 (S1 and S2), and the receptor-binding domain (RBD). Median fluorescence intensity (MFI) values were acquired using a Flexmap 3D reader, with a minimum of 50 microspheres per analyte and well. In addition to SARS-CoV-2, antibody responses were measured against multiple herpesviruses (plasma dilution 1/500), including cytomegalovirus (CMV; pp150, ab43039, Abcam), Epstein-Barr virus [EBV; early antigens diffuse (EAD, ebv-272-a, Prospecbio), EBV nuclear antigen (EBNA, ebv-276-a, Prospecbio), viral capsid antigen p18 (VCA-p18,ebv-273-a, Prospecbio), and ZEBRA (MBS505029, Mybiosource)], human herpesvirus 6A (HHV-6A, CSB-EP345592HJZ-100ug, Bionova), herpes simplex virus type 1 (HSV-1, ab43048, Abcam), and varicella-zoster virus glycoprotein E (VZV gE, vzv-231-a, Prospecbio).

Plasma neutralizing capacity was assessed via a pseudovirus-based assay against the ancestral virus (WH-1) as described previously (19).

### 2.5 Statistical and bioinformatic analyses

Continuous variables are presented as medians with interquartile ranges (IQR), and categorical variables as counts and percentages. Comparisons between study groups (uninfected, recovered, and LC, or symptom-based LC clusters) were performed using Kruskal-Wallis tests for continuous variables and Fisher’s exact tests for categorical variables. *Post-hoc* pairwise comparisons were performed using Dunn’s tests with Holm adjustment for multiple pairwise comparisons where appropriate. P-values of exploratory analyses with multiple testing were corrected using false discovery rate (FDR) method. FDR correction was applied separately within each predefined analytical family, with between-group and LC cluster-level analyses treated independently because they addressed distinct hypotheses. Missing data were not coded as negative values, and percentages were calculated using available observations as denominators. Standardized mean differences (SMD) were also reported as a complementary metric to p-values. To assess the robustness of the findings to uncertainty in acute infection ascertainment, the principal analyses were repeated after excluding participants whose initial SARS-CoV-2 infection was based solely on clinical diagnosis.

We used hierarchical clustering analysis as previously described (20) to group participants with LC based on their presenting symptoms at the time of sampling including all PWLC (N = 643) from the KING cohort extension. Silhouette width analysis was performed to study the optimal number of clusters, which was selected according to it, alongside clinical relevance criteria, and clinical and demographic characteristics were compared across clusters.

For Luminex-derived soluble immune biomarkers, values below the lower limit of detection were replaced by one-half of the assay-specific lower limit, values above the upper limit of detection were assigned the corresponding upper limit, and values were log10-transformed before normalization. Markers with >25% undetectable values (IL-4, IL-5, IL-17A, IL-15, VEGF-A, FGF-2, MIP-1α, MIG), with similar distribution across all groups, were excluded. Batch effects were corrected using multi-MA normalization implemented in the MDimNormn R package (21). Twenty-two normalized Luminex markers were used for PCA; TGF-β was analyzed separately, included in the biomarker-testing family, and was not included in the MDimNormn-normalized PCA. Endothelial markers were log10-transformed before analysis. To evaluate the potential influence of time since symptom onset on biomarker levels, Pearson correlation analyses were performed using log10-transformed biomarker concentrations, with Spearman correlations performed as a rank-based sensitivity analysis. Correlation coefficients, 95% confidence intervals, and FDR-adjusted p-values for each biomarker are reported in **Supplementary Figure 1**.

Associations of soluble immune and endothelial markers with infection and symptom persistence was evaluated using linear regression models in which infection and persistence were represented as separate binary variables. The primary models included age, sex, and hospitalization during acute COVID-19 as covariates. A sensitivity analysis additionally included obesity in participants with available data.

Plasma SARS-CoV-2 RNA detection was compared between recovered participants and PWLC using Fisher’s exact test. Ct values for the N target were compared among RNA-positive participants using two-sided Wilcoxon rank-sum tests. Ct values were interpreted as assay measurements and not calibrated estimates of viral load. Cluster-level RNA-detection comparisons used Fisher-Freeman-Halton tests.

For each isotype-antigen combination of SARS-CoV-2 antibodies, seropositivity thresholds were calculated from uninfected control samples. MFI values were log10-transformed, and the cut-off was defined as the mean plus three standard deviations on the log10 scale, followed by back-transformation to the original MFI scale. Fisher’s exact test (with FDR corrected p-values) assessed intergroup positivity rates. Antibody responses to other viruses were analyzed using Kruskal-Wallis tests with FDR correction.

For EBV, CMV, HSV-1, and HHV-6A antibodies, the absence of confirmed seronegative controls precluded the use of standard positivity thresholds; therefore, two complementary exploratory analytical approaches were applied to characterize serological profiles potentially associated with viral reactivation or latency. Antibody intensity values (MFI) were first log10-transformed and assumed to follow an approximately normal distribution. For each marker, robust estimates of the mean and standard deviation were obtained using Huber’s M-estimator (22) (MASS, R) and individual Z-scores and percentiles were derived from the corresponding cumulative normal distribution. For each participant, marker-specific percentiles were averaged separately for antibodies associated to viral reactivation or latency to calculate a composite percentile score that summarized the relative magnitude of each antibody profile. For most herpesviruses, IgA and IgM responses were considered reactivation-associated, whereas IgG responses were considered latency-associated. For EBV, classification was based on antigen-specific biology, with anti-EAD IgM/IgA and anti-ZEBRA IgM/IgA considered reactivation-associated, whereas anti-EBNA IgG and persistently detectable anti-VCA-p18 IgG were classified within the latency-associated category for analytical purposes, reflecting established infection rather than ongoing lytic activity. Exploratory reactivation-associated antibody indexes were subsequently calculated as the mean Z-score of reactivation-associated antibody MFIs minus the mean Z-score of latency-associated antibody MFIs.

Random forest models were used to explore the ability of pre-specified biomarker panels to discriminate between study groups. The differences in sample size of the groups were addressed through the use of the cutoff parameter, and the number of trees was set to 500. Performance was evaluated using repeated stratified five-fold cross-validation with 20 repeats. The mtry parameter was tuned within each training set using out-of-bag balanced accuracy. Out-of-fold discrimination and classification performance were summarized using ROC AUC, balanced accuracy, sensitivity, specificity, and class-specific metrics, with participant-level bootstrap 95% confidence intervals (2,000 bootstraps). Variable-importance measures were summarized across resamples and were interpreted as exploratory rather than inferential evidence.

Associations between biomarkers and persistent symptoms were explored among PWLC using penalized logistic regression models with elastic-net regularization (alpha = 0.5; glmnet package, R). Candidate outcomes were binary symptoms with at least 20 participants with and 20 without the symptom. Models included standardized biological predictors, sex, age, hospitalization during acute COVID-19, and detectable plasma SARS-CoV-2 RNA. Performance was evaluated by repeated nested cross-validation, with five outer folds repeated twenty times. Within each outer training set, the penalty parameter lambda was selected by inner cross-validation to maximize ROC AUC. Out-of-fold predicted probabilities were used to estimate ROC AUC and other classification metrics; 95% confidence intervals were obtained by 2,000 participant-level bootstrap resamples. Complete coefficients for all candidate predictors and eligible symptom models, including coefficients shrunk to zero, are provided in **Supplementary Data Sheet 1.**

All statistical analyses were performed using R (version 4.6.1). All tests were two-sided. FDR-adjusted p-values <0.05 were considered statistically significant, and FDR-adjusted p-values from 0.05 to 0.1 were considered suggestive exploratory findings.

## 3 Results

### 3.1 Cohort characteristics

The study included 312 participants: 139 PWLC, 99 recovered, and 74 uninfected controls (**Figure 1**). Demographic and clinical characteristics are summarized in **Table 1**. Recovered individuals were older than both PWLC and uninfected participants [median (IQR), 53 (42-62), 46 (38-54) and 42 (32-53), respectively, p<0.001]. The proportion of women was higher in LC group (71.2%) compared to recovered (41%) and uninfected (64%) participants (p<0.001). Time from symptom onset was slightly longer in PWLC than in recovered individuals [median 8.1 (6.7-9) months vs 7.2 (6.4-8.3) months; p=0.003]. Hospitalization during acute COVID-19 occurred less frequently among PWLC (40.3% vs 53.5%; p=0.048), while there were no differences in ICU entry or oxygen needs between groups. The relatively high percentage of hospitalized participants in both groups is related to the inclusion of unvaccinated participants infected during the first pandemic wave, when hospitalization thresholds and disease severity differed from later phases of the pandemic. All participants were unvaccinated at the time of sampling, and almost all infections occurred during the early pandemic waves (preceding Alpha emergence). Several comorbidities differed between groups, with allergies and obesity being more frequent in PWLC, whereas dyslipidemia and ischemic heart disease were less prevalent (p<0.05 in all cases).

**Table 1:** Participant characteristics.

Variable	Uninfected N = 74		Recovered N = 99		LC N = 139		*P-value*	*Pairwise Holm-adjusted* *P-values*			*Absolute standardized mean differences*		
	Value	Missing	Value	Missing	Value	Missing	*Overall*	U vs R	U vs LC	R vs LC	U vs R	U vs LC	R vs LC
Age, years, median [IQR]^1^	42 [32.2–52.8]	0	53 [42–62]	0	46 [38–53.5]	0	***<0.001***	***<0.001***	*0.141*	***0.008***	0.561	0.223	0.356
Female sex, N (%)^2^	47 (63.5)	0	41 (41.4)	0	99 (71.2)	0	***<0.001***	***0.011***	*0.279*	***<0.001***	0.454	0.165	0.63
Time from symptoms onset to sample collection, months, median [IQR]^3^	–	–	7.2 [6.4–8.3]	0	8.1 [6.7–9]	0	***0.003***	*–*	*–*	***0.003***	*–*	*–*	0.366
Hospitalization for acute COVID-19, N (%)^2^	–	–	53 (53.5)	0	56 (40.3)	0	***0.048***	*–*	*–*	***0.048***	*–*	*–*	0.268
ICU/RICU entry for acute COVID-19, N (%)^2^	–	–	7 (7.1)	0	13 (9.4)	0	*0.639*	*–*	*–*	*0.639*	*–*	*–*	0.083
COVID-19 Severity, N (% of hospitalized)^2^							*0.451*			*0.451*			
High-flow oxygen	–	–	9 (17.0)	0	15 (26.8)	0					*–*	*–*	0.239
Low-flow oxygen	–	–	27 (50.9)	0	26 (46.4)	0					*–*	*–*	0.09
No oxygen requirement	–	–	14 (26.4)	0	12 (21.4)	0					*–*	*–*	0.117
Diagnosis method, N (%)^2^		–		0		0	*<0.001*	*–*	*–*	*<0.001*	*–*	*–*	0.129
Nasopharyngeal SARS-CoV-2 PCR	–		68 (68.7)		87 (62.6)								
Lateral Flow Rapid Test	–		3 (3.0)		1 (0.7)								
Serology	–		25 (25.3)		24 (17.3)								
Clinical diagnosis	–		3 (3.0)		27 (19.4)								
SARS-CoV-2 Vaccination, N (%)^2^	0 (0.0)	0	0 (0.0)	0	0 (0.0)	0	*1*			*1*	0	0	0
SARS-CoV-2 variant, N (%)^2^		–		0		0	*1*			*1*			0.009
Pre-Alpha	–		96 (97.0)		135 (97.1)								
Alpha	–		3 (3.0)		4 (2.9)								
Comorbidities, N (%)^2^													
High blood pressure	4 (14.8)	47	25 (34.2)	26	27 (21.3)	12	*0.065*	*0.194*	*0.6*	*0.194*	0.464	0.168	0.293
Allergy	3 (11.1)	47	20 (27.0)	25	47 (37.0)	12	***0.02***	*0.225*	***0.035***	*0.225*	0.414	0.636	0.215
Diabetes	1 (3.7)	47	13 (17.6)	25	12 (9.4)	12	*0.112*	*0.314*	*0.467*	*0.314*	0.462	0.233	0.239
Obesity	1 (3.8)	48	14 (19.2)	26	38 (30.4)	14	***0.005***	*0.191*	***0.009***	*0.191*	0.495	0.753	0.262
Dyslipidemia	7 (25.9)	47	31 (41.9)	25	30 (23.6)	12	***0.023***	*0.338*	*0.807*	***0.032***	0.342	0.053	0.397
Ischemic heart disease	2 (7.4)	47	6 (8.1)	25	1 (0.8)	12	***0.009***	*1*	*0.159*	***0.032***	0.026	0.339	0.361
Headache	2 (7.4)	47	5 (6.8)	25	14 (11.0)	12	*0.644*	*1*	*1*	*1*	0.025	0.125	0.15
Arrhythmia	2 (7.4)	47	6 (8.1)	25	8 (6.3)	12	*0.812*	*1*	*1*	*1*	0.026	0.044	0.07
Thromboembolic disease	1 (3.7)	47	1 (1.4)	25	1 (0.8)	12	*0.349*	*0.963*	*0.963*	*1*	0.15	0.198	0.055
Stroke	0 (0.0)	47	1 (1.4)	25	0 (0.0)	12	*0.443*	*1*		*0.736*	0.166	0	0.166
Heart failure	0 (0.0)	47	1 (1.4)	25	0 (0.0)	12	*0.443*	*1*		*0.736*	0.166	0	0.166

^1^ Kruskal-Wallis test with pairwise Dunn’s post-hoc comparisons; ^2^Fisher’s exact test; ^3^Wilcoxon rank-sum test.

Significant p-values are indicated in bold.

PWLC were classified into three symptom-based clusters through unsupervised hierarchical clustering (**Supplementary Figure 2**; **Supplementary Table 2**). Cluster A represented the mildest LC phenotype, mainly characterized by fatigue and dyspnea, whereas cluster C showed the highest symptom burden, with a median of 17 [14-19] simultaneous symptoms affecting multiple organ systems. Cluster B represented an intermediate phenotype between clusters A and C, characterized by the co-occurrence of fatigue, dyspnea, neurocognitive complaints, myalgia, and headache. The proportion of women increased from the mildest phenotype to the phenotype with the greatest number of concurrent symptoms (60.4% in cluster A, 65.8% in cluster B, and 87.5% in cluster C; p=0.006) (**Supplementary Table 2**).

### 3.2 Immune dysregulation and endothelial activation in LC

We next evaluated circulating immune and endothelial biomarkers across study groups. Overall, soluble marker profiles showed substantial overlap between groups, with only limited differences associated with LC. No meaningful correlations were observed between biomarker concentrations and time from symptom onset within the 5–14 months post-infection interval (all Pearson R < 0.2 and FDR > 0.1; **Supplementary Figure 1**), indicating that the observed biomarker profiles were not driven by differences in sampling time. Among the analyzed biomarkers, IL-1 receptor antagonist (IL-1RA) showed the strongest association with LC, displaying progressively higher levels from uninfected to recovered and PWLC groups (uninfected vs recovered, p=0.002; uninfected vs PWLC, p<0.001; recovered vs PWLC, p<0.001, **Figure 2A**). In contrast, IL-1β levels were higher in recovered individuals than in uninfected controls (p=0.005), whereas PWLC showed a trend towards higher IL-1β compared with uninfected controls (p=0.093, **Figure 2B**). Consequently, the IL-1β/IL-1RA ratio progressively decreased from uninfected to recovered and PWLC groups (uninfected vs recovered, p=0.005; uninfected vs PWLC, p <0.001; recovered vs PWLC, p <0.001, **Figure 2C**). Soluble P-selectin levels were also significantly elevated in PWLC compared to uninfected controls (p=0.009) and showed higher, but not significant levels than in recovered individuals (**Supplementary Figure 3A**), supporting persistent endothelial or thromboinflammatory perturbations.

**Figure 2:**
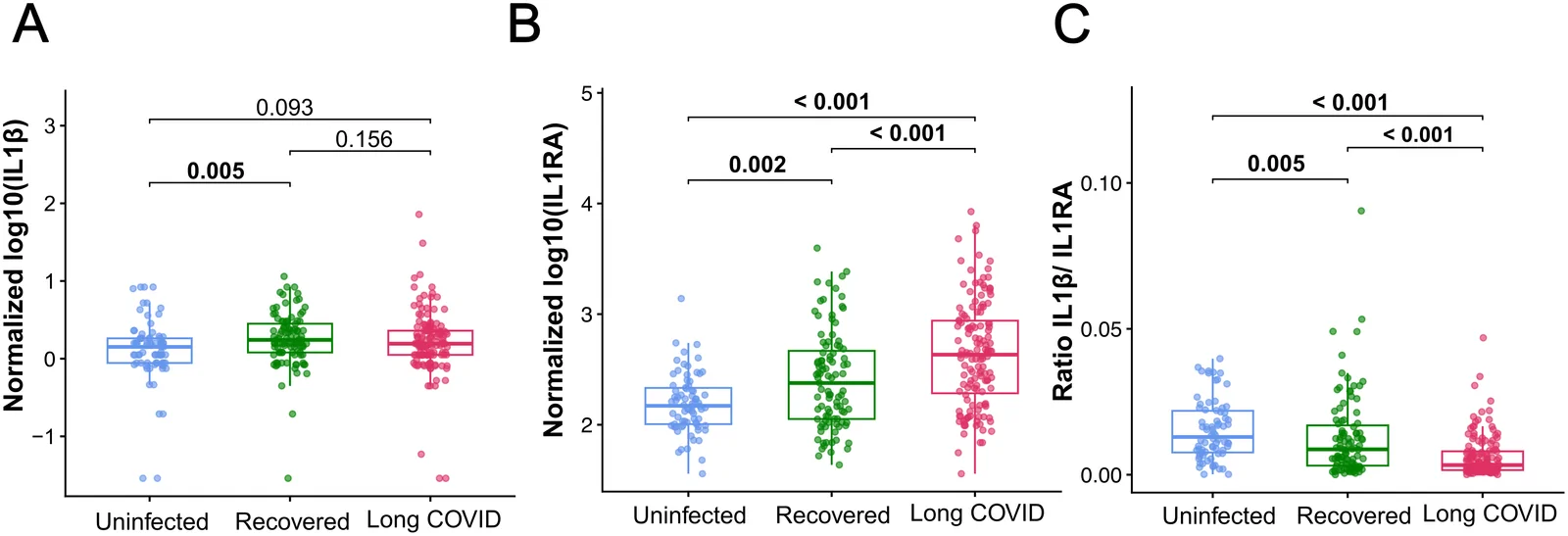
IL-1β/IL-1RA alterations. Batch-normalized log10-transformed IL-1β **(A)** and IL1RA **(B)** levels, and IL-1β/IL-1RA ratio **(C)** calculated from raw non-normalized concentration values. Statistical comparisons between groups were performed using Kruskal–Wallis tests followed by Dunn’s *post-hoc* tests with Holm adjustment for multiple pairwise comparisons. Center lines represent medians, boxes indicate interquartile ranges, and whiskers represent 1.5× the interquartile range.

Additional soluble markers differed primarily according to prior SARS-CoV-2 infection rather than symptom persistence (**Supplementary Figure 3A**). HGF, RANTES, ANG-1, and ANG-2 levels were increased in previously infected individuals compared with uninfected controls (p<0.03 for all comparisons between uninfected and infected groups), whereas IP-10 levels were reduced in PWLC compared to the uninfected group (p=0.0085) but did not differ from those in the recovered group. In contrast, IL-7 levels were higher in recovered participants than in both LC and uninfected groups (p=0.031 and p=0.001, respectively). In multivariable models including infection, symptom persistence, age, sex, and hospitalization, IL-1RA was the only soluble marker associated with symptom persistence (p=0.001, **Table 2**). In contrast, although P-selectin differed between PWLC and uninfected participants in the unadjusted analysis (p=0.022), it was not associated with symptom persistence after FDR adjustment (p=0.14). Other biomarker associations predominantly reflected prior infection or clinical and demographic covariates: RANTES, IL-7, ANG-1, ANG-2, and HGF were associated with prior infection, IL-1RA and HGF with hospitalization, and IL-12 and ET-1 with sex (**Table 2**). In a sensitivity model additionally adjusting for obesity (**Table 2**), the association between IL-1RA and symptom persistence remained nominally significant (p=0.009) but did not remain significant after correction for multiple testing (FDR-adjusted p=0.206). Hospitalization and obesity were also nominally associated with IL-1RA levels, but neither association remained significant after FDR correction (**Table 2**). Together, these findings indicate the direction and nominal association between IL-1RA and symptom persistence were maintained after adjustment for obesity, although this association did not remain statistically significant after FDR correction.

**Table 2:** Multivariate linear regression of soluble markers adjusted by age, sex and hospitalization.

Biomarker	KW FDR p-value	Significant Dunn-Holm comparisons^1^	Model: infection + persistence + age + sex + hospitalization – N = 293				Model: infection + persistence + age + sex + hospitalization + obesity – N = 224			
			Term	Adjusted β (95% CI)	p-value	p-value FDR	Term	Adjusted β (95% CI)	p-value	p-value FDR
IL1RA	**1.69E-09**	LC>U; LC>R; R>U	Persistence	0.23 (0.11 – 0.34)	**0.0001**	**0.003**	Persistence	0.19 (0.05 – 0.33)	**0.009**	0.206
			Hospitalization	0.20 (0.07 – 0.32)	**0.002**	**0.022**	Hospitalization	0.16 (0.01 – 0.30)	**0.032**	0.299
							Obesity	0.15 (0.004 – 0.29)	**0.044**	0.504
P-selectin	**0.048**	LC>U	Sex	0.05 (0.008 – 0.09)	**0.018**	0.089	Sex	0.06 (0.01 – 0.11)	**0.011**	**0.057**
			Persistence		**0.022**	0.150				
RANTES	0.109	LC>U; R>U	Infection	0.05 (0.01 – 0.08)	**0.005**	**0.043**	Infection	0.06 (0.01 – 0.10)	**0.009**	**0.044**
IL-7	**0.020**	R>LC; R>U	Infection	0.18 (0.05 – 0.30)	**0.005**	**0.043**	Infection	0.28 (0.11 – 0.45)	**0.001**	**0.023**
ANG-1	**3.66E-08**	LC>U; R>U	Infection	0.15 (0.04 – 0.25)	**0.005**	**0.027**	Infection	0.18 (0.05 – 0.31)	**0.008**	**0.042**
ANG-2	**6.64E-05**	U>LC; U>R	Infection	-0.10 (-0.18 – -0.01)	**0.024**	0.059	Infection	-0.15 (-0.27 – -0.03)	**0.017**	**0.043**
HGF	**1.57E-08**	LC>U; R>U	Hospitalization	0.09 (0.04 – 0.15)	**0.001**	**0.022**	Hospitalization	0.07 (0.004 – 0.14)	**0.039**	0.299
			Infection	0.10 (0.03 – 0.17)	**0.006**	**0.043**	Infection	0.13 (0.03 – 0.23)	**0.009**	**0.044**
IP-10	0.066	U>LC	Age	0.004 (0.002 – 0.006)	**0.001**	**0.011**	Age	0.005 (0.003 – 0.008)	**1.09E-05**	**2.50E-04**
							Infection	-0.12 (-0.22 – -0.02)	**0.024**	**0.091**
IL12/IL23p40	**0.001**	-	Sex	-0.10 (-0.16 – -0.04)	**0.001**	**0.027**	Sex	-0.13 (-0.20 – -0.06)	**0.000**	**0.011**
Eotaxin	**0.001**	LC>U; R>U	Age	0.004 (0.002 – 0.007)	**0.001**	**0.011**	Age	0.004 (0.0007 – 0.007)	**0.016**	0.186
							Obesity	-0.09 (-0.18 – -0.004)	**0.041**	0.504
ET-1	0.185	-	Age	0.004 (0.003 –0.005)	**7.74E-08**	**3.87E-07**	Age	0.004 (0.003 – 0.006)	**1.98E-06**	**9.91E-06**
MCP1	**0.036**	LC>U; R>U	-				-			
TGF-b	**0.001**	R>U; LC>U	-				-			
IFNg	**0.070**	R>U	-							

^1^ LC, Long COVID; R, Recovered; U, uninfected, FDR: False discovery rate.

Variables with p-adj < 0.1 are shown. P-values were corrected for multiple testing using FDR. For sex, reference group was Female. For other binary predictors, the reference value was always “No”.

Significant p-values are indicated in bold.

Consistent with these findings, principal component analysis showed substantial overlap in the overall biomarker profiles across study groups (**Supplementary Figure 3B**).

### 3.3 Circulating SARS-CoV-2 RNA and antiviral immunity

SARS-CoV-2 RNA was detected in plasma from a minority of both PWLC and recovered participants (7.2% and 4.3%, respectively, p=0.414) (**Figure 3A**). All participants with detected viral RNA were positive for N gene, except for two PWLC, who were positive for E gene, and both N and E genes, respectively. Among participants with detectable plasma SARS-CoV-2 RNA, Ct values tended to be lower in PWLC than in recovered individuals (Mann–Whitney p=0.08, **Figure 3B**).

**Figure 3:**
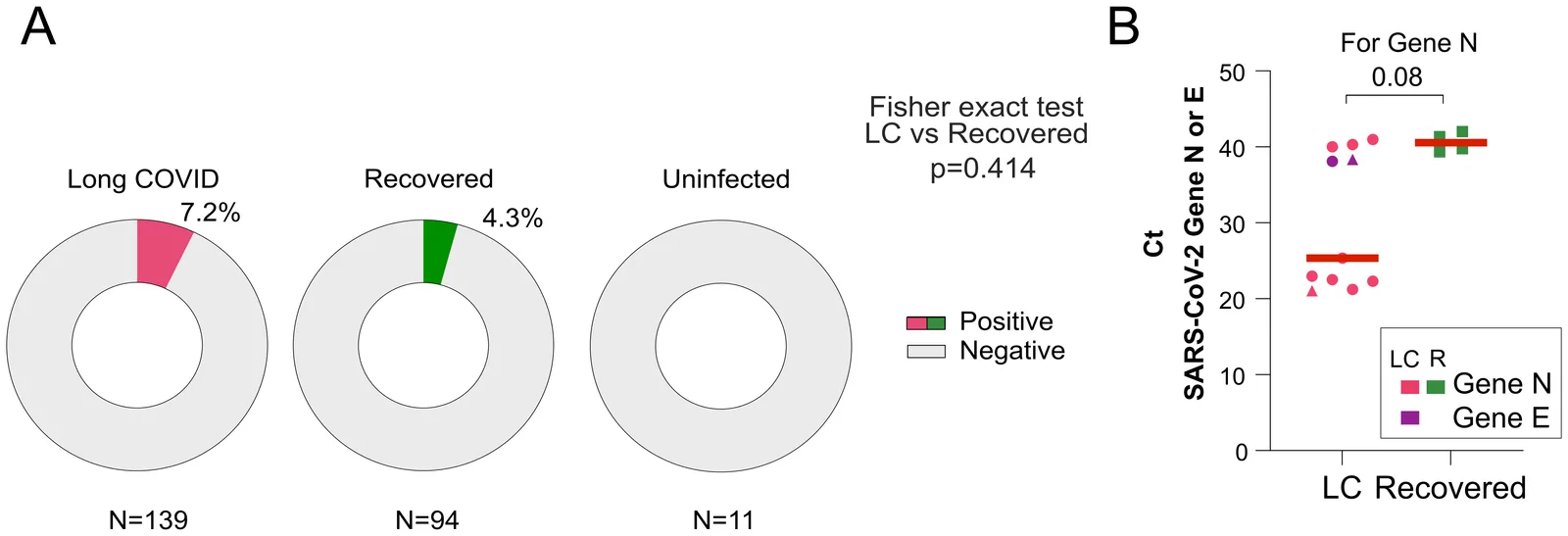
Detection of plasma SARS-CoV-2 RNA. **(A)** Proportion of participants with detectable SARS-CoV-2 RNA in plasma from uninfected, recovered and PWLC, for either N or E genes. Statistical comparisons between LC and recovered participants was performed using Fisher’s exact test. **(B)** Cycle threshold (Ct) values among participants with detectable plasma SARS-CoV-2 N RNA for gene N or E gene. Lower Ct values indicate higher levels of circulating viral RNA. Lines represent medians. A triangle denotes the single PWLC sample positive for both the N and E genes.

SARS-CoV-2-specific humoral responses were broadly comparable between PWLC and recovered individuals, with similar IgG, IgA, IgM antibody levels against spike and nucleocapsid antigens, as well as comparable plasma neutralizing capacity against WH-1 SARS-CoV-2 (**Supplementary Figure 4A**). Nevertheless, recovered individuals exhibited higher overall SARS-CoV-2 seropositivity than PWLC (91.9% vs 81.6%, respectively, p=0.025), with higher positivity for SARS-CoV-2 IgG and IgA responses (p=0.008 and p=0.038, respectively), particularly against S1 and S2, whereas IgM seropositivity was similar between groups; however, these differences did not remain significant after FDR correction (**Supplementary Table 3**; **Supplementary Figure 4B**).

### 3.4 Herpesvirus reactivation-associated serological profiles

We evaluated antibody profiles associated with herpesvirus latency and reactivation across multiple viruses (EBV, CMV, VZV, HSV-1, HHV-6A). Percentile-based analyses revealed a shift toward reactivation-associated EBV antibody profiles in PWLC compared with recovered and uninfected participants (p<0.02 in both cases), with lower latency-associated EBV profiles compared with recovered and uninfected groups (p=0.024 and p=0.1 respectively, **Figure 4A**). To further characterize these findings, we analyzed individual EBV-associated antibodies. Levels of latency-associated antibodies (EBNA and VCA-p18 IgG) and most reactivation-associated antibodies (IgA and IgM against EAD and ZEBRA) were broadly comparable across groups. However, PWLC showed significantly higher levels of IgA anti-ZEBRA (an antibody response associated with EBV lytic cycle) compared to uninfected individuals (p<0.0001), with a suggestive increase compared with recovered participants (p=0.074) (**Figure 4B**). Consistently, the integrated EBV reactivation-associated antibody index was higher in PWLC than in recovered and uninfected participants (p=0.007 and p=0.035, respectively, **Figure 4C**), supporting an altered EBV-specific humoral profile in PWLC.

**Figure 4:**
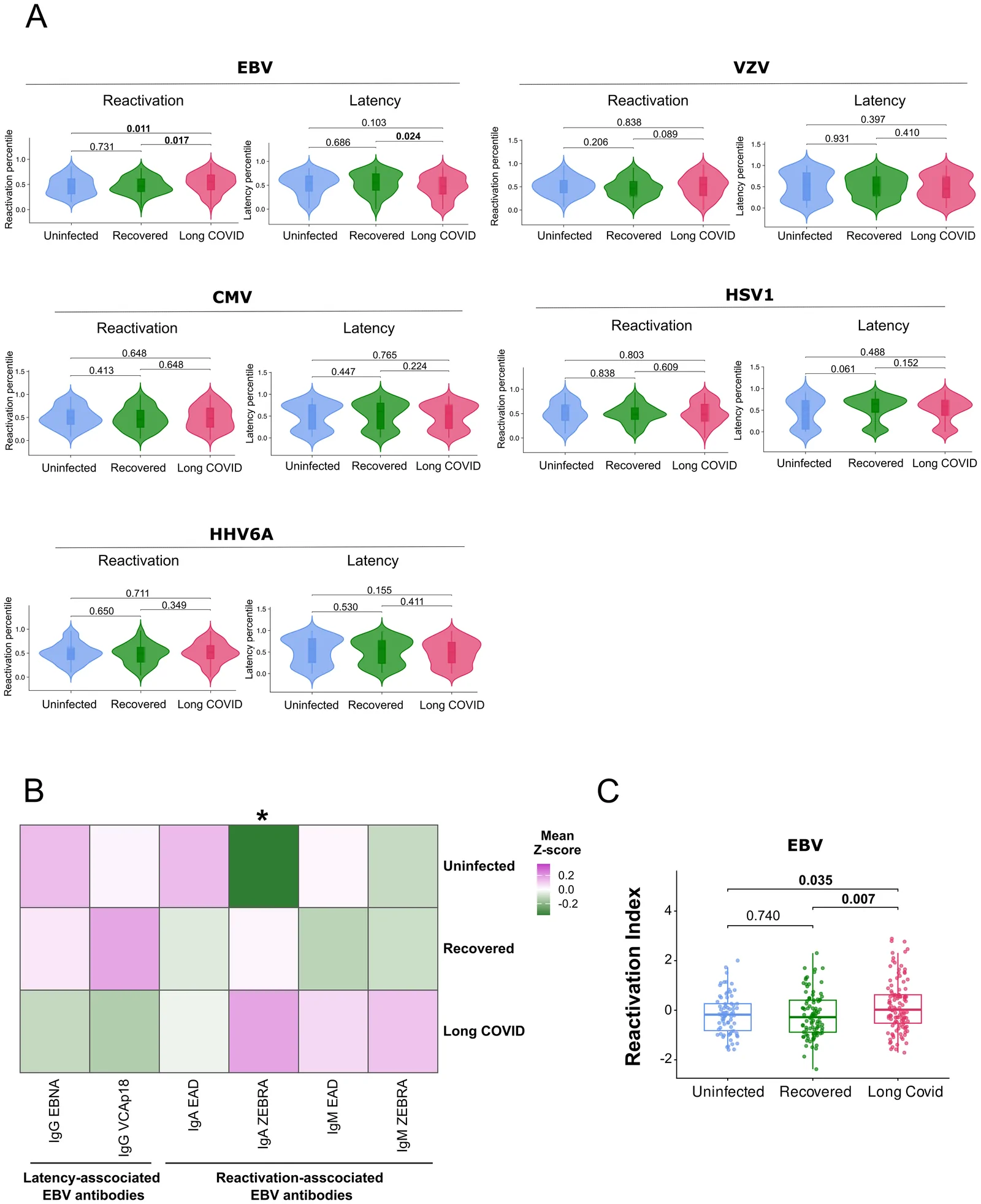
Herpesvirus reactivation-associated antibody profiles. **(A)** Percentile-based distributions of latency-associated and reactivation-associated herpesvirus antibody responses across study groups. Percentiles were calculated from log10-transformed antibody MFIs as described in the Methods section. Statistical comparisons between groups were performed using Kruskal–Wallis tests followed by Dunn’s *post-hoc* tests. **(B)** Z-scores of anti-EBV antibody responses, including latency-associated (EBNA and VCA-p18 IgG) and reactivation-associated (EAD and ZEBRA IgA/IgM) antibodies. Significant associations after Kruskal-Wallis tests followed by FDR correction are marked with *. **(C)** EBV reactivation-to-latency antibody index calculated from normalized antibody responses associated with viral latency and reactivation. Statistical comparisons between groups were performed using Kruskal–Wallis tests followed by Dunn’s *post-hoc* tests. P-values are indicated in the figure. Center lines represent medians, boxes indicate interquartile ranges, and whiskers represent 1.5× the interquartile range.

No consistent differences were observed for CMV, VZV, HHV-6A, or HSV-1 (**Figure 4A**; **Supplementary Figure 5**). Although the HSV-1 reactivation index was lower in recovered individuals than in uninfected group (p=0.003), with a trend toward lower values compared to PWLC (p=0.086).

### 3.5 Sensitivity analyses restricted to laboratory-confirmed SARS-CoV-2 infection

To confirm that the observed findings were maintained when LC was restricted to individuals with laboratory-confirmed SARS-CoV-2 infection preceding persistent symptoms, we repeated the principal analyses after excluding participants whose initial SARS-CoV-2 infection was based solely on clinical diagnosis. The restricted cohort included 112 PWLC, 96 recovered participants, and 74 uninfected controls (**Supplementary Table 4**). The principal immune, vascular, viral RNA, and EBV-associated findings were maintained, including increased IL-1RA and altered IL-1β/IL-1RA ratio in PWLC, the P-selectin group difference, low and comparable frequencies of detectable plasma SARS-CoV-2 RNA in PWLC and recovered participants (6.2% vs 4.3%; p=0.758), and the EBV reactivation-associated serological patterns (**Supplementary Figure 6**). In contrast, differences in SARS-CoV-2-specific seropositivity were no longer observed after restricting the analysis to laboratory-confirmed infections. Overall SARS-CoV-2-specific antibody positivity was comparable between PWLC and recovered participants (91.1% vs 93.6%; p=0.604), with no significant differences in IgG, IgA, or IgM seropositivity. Thus, the principal immune, vascular, plasma RNA, and EBV-associated patterns were maintained after exclusion of clinically diagnosed infections, whereas the SARS-CoV-2-specific seropositivity differences were not reproduced.

### 3.6 Integrated biomarker analysis using random forest classification

To determine whether the biological alterations identified across individual analyses could collectively discriminate clinical groups, we integrated the measured biomarkers using random forest classification. Models were evaluated using progressively broader predictor panels comprising immune biomarkers, immune and vascular biomarkers, and an integrated multimodal panel incorporating immune and vascular biomarkers together with SARS-CoV-2-specific humoral responses, herpesvirus serology, and plasma SARS-CoV-2 RNA.

Classification performance was higher for distinguishing previously infected from uninfected participants than for distinguishing PWLC from recovered participants (**Supplementary Figure 7A**; **Supplementary Table 5**). Using the immune and vascular biomarker panel, discrimination between infected and uninfected participants reached a ROC AUC of 0.820 (95% CI, 0.759–0.876) and balanced accuracy of 0.735 (0.676–0.788), whereas discrimination between PWLC and recovered participants was substantially lower (ROC AUC, 0.645 [0.575–0.715]; balanced accuracy, 0.627 [0.567–0.691]). Three-group classification showed intermediate performance (ROC AUC, 0.750 [0.709–0.789]; balanced accuracy, 0.666 [0.625–0.704]).

To directly assess whether expanding the predictor panel improved discrimination between PWLC and recovered participants, all three panels were additionally compared in the same subset of 220 participants using identical cross-validation folds. ROC AUCs were 0.599 (95% CI, 0.519–0.677) for the immune panel, 0.604 (0.528–0.683) after the addition of vascular biomarkers, and 0.633 (0.558–0.707) for the integrated multimodal panel. The corresponding paired differences in ROC AUC were small, and their 95% CIs included zero for all comparisons, as did the paired differences in accuracy, balanced accuracy, sensitivity, and specificity (**Supplementary Table 5C**). Thus, progressively expanding the biomarker panel provided no clear improvement in discrimination between PWLC and recovered participants, supporting the absence of a clearly discriminative LC profile among the circulating biomarkers evaluated.

Permutation-importance analyses identified IL-1RA, ANG-1, and HGF among the most influential predictors in the three-group and infected-versus-uninfected models using the immune and vascular biomarker panel (**Supplementary Figure 7B**). In the multimodal PWLC-versus-recovered model, EBV reactivation-associated antibody percentile, anti-RBD IgM, and IL-1RA showed the highest permutation importance. Notably, IL-1RA ranked among the most influential predictors across all three classification tasks, consistent with its recurrent signal in the preceding analyses. However, variable-importance estimates were considered exploratory and do not establish independent associations.

### 3.7 Biomarkers associated to persistent symptom burden

To explore biological patterns associated with individual persistent symptoms, elastic-net penalized logistic regression models were fitted among 135 PWLC with complete predictor data. Of the 36 symptoms evaluated, 25 met the prespecified eligibility criterion of at least 20 participants with and without the symptom. Overall discrimination was limited (median out-of-fold ROC AUC, 0.54; range, 0.39-0.70), with the highest ROC AUC observed for fatigue (0.70; 95% CI, 0.50-0.81) and low-grade fever (0.69; 95% CI, 0.59-0.79) (**Figure 5**; **Supplementary Table 6**; **Supplementary Data Sheet 1**). Using the prespecified stability criteria of ≥75% selection frequency and ≥90% sign consistency, recurrent predictors were identified in 24 of the 25 eligible symptom models and encompassed immune, endothelial, and virus-specific antibody measurements. IL-1RA was the most recurrent predictor, being positively retained in seven symptom models, followed by IL-8 in six models; ET-1, TGF-β, IgA anti S, IgG anti CMV pp150, and IgG anti HHV-6A were each retained in five models (**Figure 5**). These heterogeneous patterns and the generally modest model performance suggest partially overlapping symptom-associated biological profiles, which should be interpreted as exploratory rather than as validated predictive signatures.

**Figure 5:**
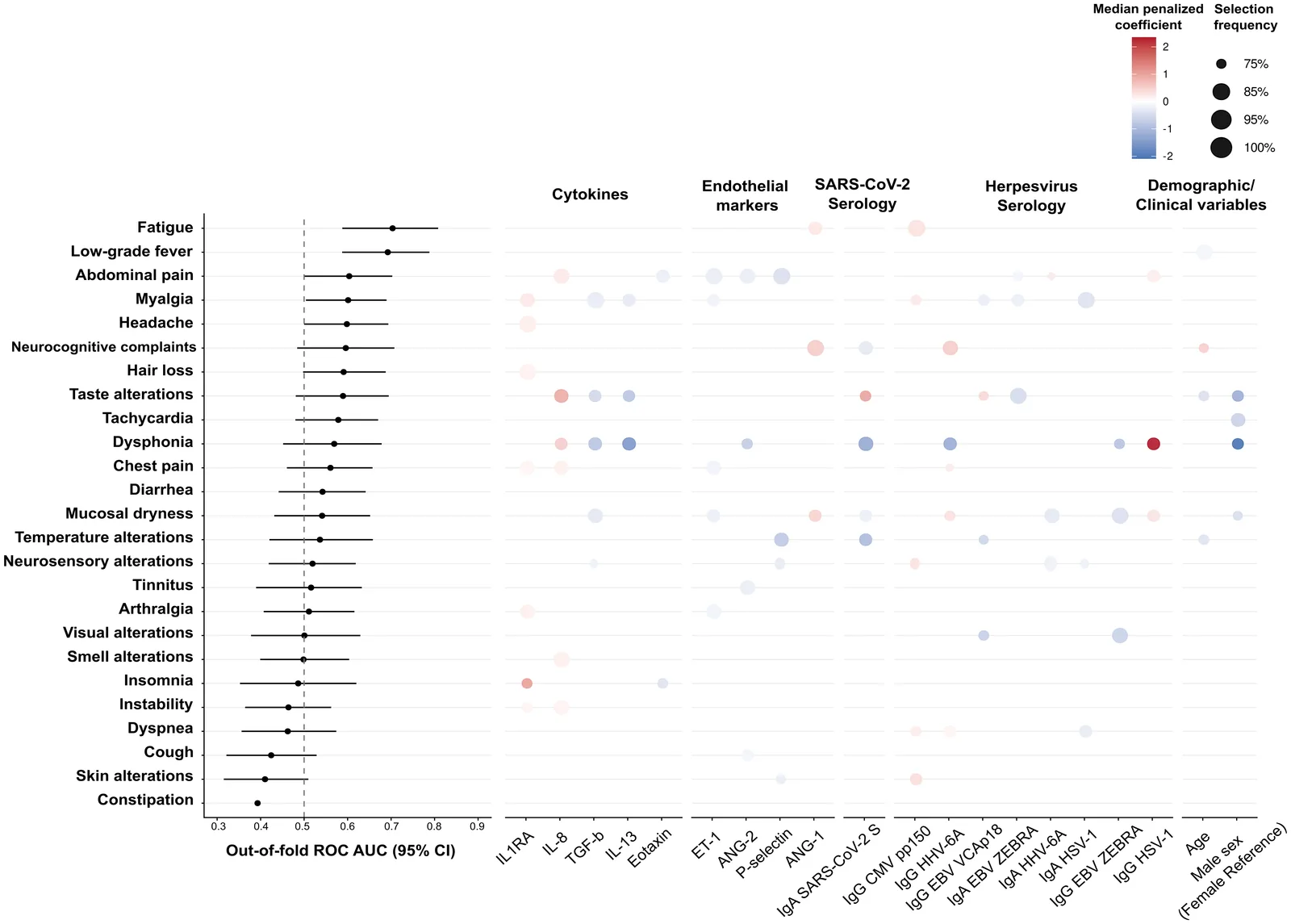
Out-of-fold discrimination and recurrent predictors of individual long COVID symptoms. Each row represents an eligible persistent symptom model. The left panel shows the out-of-fold receiver operating characteristic area under the curve (ROC AUC) with 95% confidence intervals obtained from repeated nested cross-validation. The right panel displays the 20 predictors most frequently retained across symptom models after elastic-net regularization. Point size represents the frequency with which a predictor was selected across outer resampling fits, and color indicates the median penalized coefficient among fits in which that predictor was selected (red-positive, blue-negative). Only symptom-predictor pairs meeting the prespecified visualization criteria of selection frequency ≥75% and sign consistency ≥90% are shown.

We next evaluated biological differences across the three symptom-based LC clusters (A, B, C). Participants in cluster C, characterized by the highest symptom burden, tended to show higher IL-1RA levels than clusters A and B, although differences were not statistically significant (C vs A, p=0.14; C vs B, p=0.08, **Supplementary Figure 8A**). Consistent with this pattern, the IL-1β/IL-1RA ratio tended to be lower in cluster C than in cluster A (p=0.056), while no differences were observed between the other clusters (**Supplementary Figure 8A**). Similarly, P-selectin levels were lower in cluster C compared with both cluster A (p=0.082) and cluster B (p=0.011) (**Supplementary Figure 8A**). Because the proportion of women increased across symptom clusters, particularly in cluster C, we repeated the cluster biomarker analyses using multivariable models adjusted for age, sex and hospitalization. No global association between cluster membership and any soluble immune or endothelial biomarker remained statistically significant after FDR correction (**Supplementary Table 8**). Results were materially unchanged after additional adjustment for obesity in the subset with available data. The proportion of participants with detectable SARS-CoV-2 RNA increased across clusters, ranging from 1.9% (1/53) in cluster A, 5.3% (2/38) in cluster B and 15.2% (7/48) in cluster C (p=0.039, **Supplementary Figure 8B**). Given the small number of RNA-positive participants, this finding should be considered exploratory. In contrast, anti-SARS-CoV-2 antibody levels and neutralizing activity did not differ between clusters (data not shown). Finally, analysis of herpesvirus reactivation-associated serological indexes revealed higher EBV and VZV indexes in clusters A and C compared with cluster B, which showed values similar to those of uninfected and recovered individuals (EBV: cluster C vs B, p=0.012; cluster A vs B, p=0.077; VZV: cluster A vs B, p=0.014 and cluster C vs B, p=0.033, **Supplementary Figure 8C**). No differences were observed for CMV or HSV-1, whereas HHV-6A reactivation-associated serological index was significantly higher in cluster A (A vs B p=0.012; A vs C p=0.008).

## 4 Discussion

In this well-characterized cohort of unvaccinated individuals following primary SARS-CoV-2 infection, LC was associated with modest alterations in immune and endothelial biomarkers and viral pathways rather than with a single discrete biomarker-defined signature. Importantly, most soluble biomarker differences primarily distinguished previously infected individuals from uninfected controls, whereas IL-1RA remained associated with persistent symptoms after adjustment for prespecified confounders, although this association did not remain significant after FDR correction in the sensitivity analysis additionally adjusting for obesity. P-selectin differed at the group level but was not independently associated with symptom persistence after adjustment. In parallel, detectable plasma SARS-CoV-2 RNA was uncommon and detected at similar frequencies in PWLC and recovered participants, while PWLC showed lower EBV latency-associated and higher reactivation-associated serological responses. Consistent with these findings, integrated random forest models discriminated prior infection more effectively than LC status, and expansion to multimodal predictor panels did not clearly improve discrimination between PWLC and recovered participants. Together, these findings support a model in which LC arises from partially overlapping biological mechanisms rather than from a dominant or uniform inflammatory phenotype.

The IL-1 axis findings are consistent with a model in which LC is characterized not by systemic hyperinflammation, but by persistent alterations in IL-1 pathway regulation. Increased IL-1RA levels together with a reduced IL-1β/IL-1RA ratio followed a progressive gradient across groups, with intermediate alterations in recovered individuals and the greatest changes in PWLC, suggesting persistent dysregulation of the IL-1 axis beyond acute infection (Rodrigues et al., 2020) and independent of COVID-19 severity. Higher IL-1RA levels in the absence of a corresponding increase in IL-1β in PWLC may reflect a compensatory attempt to restrain prolonged IL-1-driven signaling after infection. This interpretation is supported by previous studies reporting persistent elevations of IL1 family cytokines, including IL-1β, in PWLC (23). More broadly, dysregulation of the IL-1 pathways has also been described in other chronic inflammatory disorders, where they have been associated with persistent symptoms rather than acute inflammatory escalation (24, 25). Together, these findings identify altered IL-1 pathway regulation, and particularly IL-1RA, as one of the most consistent immune correlates of symptom persistence in this cohort. In contrast, other soluble mediators, including IL-7, HGF, IP-10, and RANTES, differed mainly according to prior SARS-CoV-2 infection rather than symptom persistence, supporting the idea that infection leaves a broader and longer-lasting remodeling of immune and tissue-repair pathways even in the absence of persistent symptoms (26, 27). Interestingly, IP-10 levels were lower in PWLC than in uninfected controls. Although this differs from other studies of persistent IP-10 elevation in some LC cohorts (28), it may reflect attenuated IFN-driven immune signaling or other phenotype-specific differences in antiviral immune regulation in PWLC, potentially related to distinctive characteristics of our cohort (i.e. different clinical phenotypes or disease stage) further underscoring the biological heterogeneity of LC.

Similarly, most vascular- and tissue repair-related differences, including ANG-1, ANG-2, and HGF, reflected prior SARS-CoV-2 infection rather than symptom persistence, consistent with previous reports describing persistent vascular remodeling after infection (29, 30). P-selectin showed a more heterogeneous relationship with LC. At the group level, circulating P-selectin levels were higher in PWLC than in uninfected participants, but were not independently associated with symptom persistence after adjustment for confounders, consistent with a potential alteration in platelet-endothelial biology. Given the known role of P-selectin in leukocyte adhesion, platelet aggregation, and microvascular flow impairment (31), this finding is biologically plausible and consistent with current hypotheses linking platelet activation, fibrinolysis-resistant microclots, and microvascular dysfunction to LC symptomatology (12, 13, 32). Surprisingly, P-selectin did not increase with symptom burden and was lower in the most symptomatic cluster, arguing against a simple relationship between circulating P-selectin and LC severity. Circulating P-selectin levels have shown variable relationships with clinical severity across COVID-19 studies, and the divergent patterns observed here may reflect differences in clinical phenotype, platelet activation, soluble versus membrane-associated P-selectin, or residual confounding (33–35). Thus, rather than representing a uniform marker of persistent endothelial activation or LC severity, our findings identify P-selectin as an exploratory, potentially phenotype-associated vascular signal that warrants further investigation. Deeper analyses may be required to explore this phenomenon.

Our findings on circulating SARS-CoV-2 RNA support a similarly nuanced interpretation. Detectable viral RNA in plasma was uncommon (<10%) and was present at similar frequencies in PWLC and recovered individuals, indicating that detectable circulating SARS-CoV-2 RNA is neither frequent nor specific to LC. Among RNA-positive samples, Ct values tended to be lower in PWLC, although this difference did not reach statistical significance. Given the analytical characteristics of the nested RT-qPCR assay, including the absence of a formally established limit of detection and systematic confirmation of low-level positive samples, these findings should be interpreted as low-level amplification signals rather than definitive evidence of reproducible circulating SARS-CoV-2 RNA. However, these findings are consistent with previous studies showing that viral RNA can persist beyond the acute phase, particularly during early convalescence (15, 36). Similarly, in our previous antigenemia study (37), N protein was detected at comparably low frequencies (6.9% in PWLC, 2.1% in recovered individuals, and 2.0% in uninfected controls), closely mirroring the prevalence of plasma SARS-CoV-2 RNA despite assessing a distinct viral component. In addition, increasing evidence supports the persistence of SARS-CoV-2 RNA or proteins in tissues months after infection, where they may contribute to sustained immune activation (14, 38, 39). Thus, while detectable circulating SARS-CoV-2 RNA alone is unlikely to explain LC at the population level, it may represent one manifestation of prolonged viral material that interacts with other pathogenic mechanisms in some individuals. Importantly, the absence of detectable plasma viral RNA does not exclude persistent viral reservoirs in tissue niches that cannot be captured by plasma-based analyses.

In contrast, we did not observe meaningful differences in anti-SARS-CoV-2 antibody levels or neutralizing activity between PWLC and recovered participants, even after adjustment for relevant confounders. In the full cohort, however, a higher proportion of PWLC lacked detectable SARS-CoV-2-specific IgG and IgA responses and selected spike antigens (S1 and S2), raising the possibility of a subset of PWLC with absent or inadequate seroconversion following infection. Notably, this difference was no longer observed when the analysis was restricted to participants with laboratory-confirmed SARS-CoV-2 infection, indicating that the finding was sensitive to infection ascertainment and should therefore be interpreted cautiously. Overall, our findings are consistent with longitudinal studies reporting comparable antibody responses in PWLC and recovered individuals months after infection (40, 41), although other studies have described lower neutralizing antibody titers in PWLC (42). Similarly, a study in a vaccinated LC cohort reported incomplete seroconversion to the S2 domain together with reduced anti-S1 IgG levels and impaired neutralizing activity, suggesting that alterations in SARS-CoV-2-specific humoral immunity may also occur after vaccination (11). Differences in cohort composition, vaccination status, timing after infection, and assay methodologies may contribute to these discrepancies. Nevertheless, we cannot exclude more qualitative alterations in humoral immunity, including differences in antibody subclasses or functional properties, which were not assessed in this study, but were found to be relevant in other LC studies, as well as other conditions such as Lyme arthritis (43–45).

Among herpesviruses, EBV showed the most consistent signal. PWLC displayed higher EBV reactivation-associated antibody biomarker percentiles, lower latency-associated antibody percentiles, elevated anti-ZEBRA IgA levels, and a higher EBV reactivation-associated antibody index compared with both recovered and uninfected participants. This convergence across analytical approaches suggests an altered EBV-specific humoral profile in LC, in line with previous studies linking EBV reactivation to persistent symptoms such as fatigue and neurocognitive impairment (46–48). The increased anti-ZEBRA IgA response and reactivation-associated antibody index may be compatible with previous EBV reactivation; however, cross-sectional serology cannot establish active EBV replication or determine when such reactivation may have occurred. In the absence of longitudinal serological data or direct virological measurements such as EBV DNA, we cannot distinguish previous reactivation from persistent inter-individual differences in EBV-specific immunity. Further data support altered antiviral immune control in LC, including increased EBV lytic-specific and SARS-CoV-2-specific T-cell responses in PWLC (49). In contrast, evidence for other herpesviruses was weaker and less consistent, suggesting that EBV may represent a specific viral cofactor rather than a generalizable herpesvirus effect (50). Together with the findings of detectable plasma SARS-CoV-2 RNA, these observations support the possibility that persistent antigenic stimulation and altered immune control may interact in a subset of PWLC.

The integrative random forest analyses further supported the distinction between biological effects of prior SARS-CoV-2 infection and those specifically associated with LC. Biomarker profiles discriminated previously infected from uninfected participants substantially better than PWLC from recovered individuals. Moreover, when predictor panels were compared within the same previously infected participants, progressively adding vascular and multimodal viral and serological features provided no clear improvement in LC discrimination. These findings suggest that, among the circulating biomarkers evaluated here, much of the detectable biological signal reflects previous SARS-CoV-2 infection rather than a distinct LC-specific molecular profile. The recurrent importance of IL-1RA across classification tasks was consistent with its signal in the conventional analyses, although variable importance should be interpreted as exploratory rather than as evidence of an independent association.

The symptom-level analyses further highlighted the biological heterogeneity underlying LC. Despite marked differences in clinical manifestations, circulating biomarkers showed limited ability to discriminate individual symptoms, and no symptom was associated with a clearly distinct biological profile. Instead, recurrent predictors overlapped across symptoms and encompassed immune, endothelial, and antiviral antibody responses. Notably, IL-1RA was the most recurrent predictor, extending its association with LC at the group level to multiple symptom models and further supporting altered IL-1 pathway regulation as one of the more consistent signals in our study. Other recurrent predictors, including IL-8, ET-1, TGF-β, anti-SARS-CoV-2 specific IgA, anti-CMV pp150 IgG and anti-HHV-6A IgG antibodies, were distributed across different symptoms rather than defining symptom-specific signatures. This pattern is consistent with an emerging view of LC as a biologically heterogenous condition in which immune dysregulation, vascular alterations, viral persistence or antigenic remnants, and herpesvirus-associated responses may coexist to varying degrees across individuals (51–53). Importantly, plasma SARS-CoV-2 RNA did not emerge as a stable predictor of any symptom, arguing against a direct correspondence between detectable circulating viral RNA and specific clinical manifestations in this cohort.

The cluster-level analysis reinforced this interpretation. Increasing symptom burden was not accompanied by a corresponding biological gradient, and neither viral RNA nor herpesvirus-associated serological profiles consistently tracked with clinical severity. Thus, although several biological pathways were altered in PWLC, our data do not support their organization into clearly separable molecular subtypes corresponding to either individual symptoms or symptom-defined clinical clusters. Rather, different clinical presentations may arise from variable combinations of partially overlapping biological perturbations. This interpretation is consistent with high-dimensional immune-profiling studies describing substantial immunological heterogeneity among PWLC and with recent efforts to define LC endotypes, which have generally identified overlapping rather than uniquely symptom-specific biological features (42, 53). Such heterogeneity may also help explain why biomarker-based classification of PWLC versus recovered participants remained limited in our integrated analyses. However, because the symptom-level analyses were exploratory and lacked external validation, recurrent predictor selection should be interpreted as hypothesis-generating rather than evidence of symptom-specific mechanisms.

This study has several strengths, including the use of a homogeneous cohort of unvaccinated individuals after primary infection and the integration of multiple biological domains within the same participants. However, several limitations should be acknowledged. First, the cross-sectional design precludes causal inference. Second, despite adjustment for age, sex, and hospitalization and the additional sensitivity analysis including obesity, the study groups differed in several demographic and clinical characteristics, and residual confounding by measured or unmeasured baseline factors cannot be excluded. Third, SARS-CoV-2 RNA was assessed only in plasma, rather than in tissue-based or antigenemia analyses, potentially underestimating compartmentalized reservoirs or persistent antigenic burden (37). In addition, although the nested RT-qPCR assay was optimized for sensitive detection of low-abundance plasma SARS-CoV-2 RNA, an assay-specific analytical limit of detection was not formally established and low-level positive samples were not systematically confirmed by repeat testing or independent re-extraction. Therefore, particularly given the small number of positive samples, these low-level amplification signals require independent analytical confirmation. Fourth, acute infection was clinically rather than laboratory confirmed in a subset of participants infected during the first wave, when diagnostic testing was limited. Importantly, sensitivity analyses restricted to participants with laboratory-confirmed SARS-CoV-2 infection reproduced the principal IL-1, P-selectin, plasma viral RNA, and EBV-associated findings, reducing the likelihood that these results were driven by uncertainty in initial infection ascertainment. In contrast, differences in SARS-CoV-2 seropositivity were no longer observed in this restricted cohort, indicating that this finding was not robust to infection ascertainment. Fifth, herpesvirus- associated profiles were inferred from serological markers rather than direct viral detection, although transient or compartmentalized reactivation events may be difficult to capture through single-time-point viral measurements. In addition, symptom-level models were exploratory and lacked independent validation and should therefore be considered hypothesis-generating. Finally, while the predominantly pre-Alpha, unvaccinated cohort minimizes heterogeneity related to vaccination, reinfections, and viral variants, it may limit generalizability to contemporary LC populations. A substantial proportion of symptom variability also remains unexplained, suggesting that additional mechanisms such as autonomic dysfunction, local tissue damage, microvascular alterations, or host genetic factors may contribute.

Our findings support a model in which LC arises from convergent dysregulation across immune, endothelial, and viral pathways rather than a single dominant mechanism or clearly separable molecular subtypes. Within this framework, IL-1 signaling imbalance emerged as the strongest immune correlate, alongside more heterogeneous alterations in vascular biology involving P-selectin and EBV reactivation-associated serological signatures. Although plasma SARS-CoV-2 RNA was detected at low and similar frequencies in PWLC and recovered individuals, Ct values tended to be lower among RNA-positive PWLC samples and in PWLC with the greatest symptom burden. Together, these findings identify candidate immune, vascular, and viral-associated pathways that may contribute to LC heterogeneity and warrant further mechanistic investigation and prospective validation.

## Data Availability

The datasets presented in this study can be found in online repositories. The names of the repository and accession number can be found below: CORA RDR database (www.dataverse.csuc.cat) and can be accessed through the following link: https://doi.org/10.34810/DATA3397 under the CC BY-SA 4.0.
