# Supplementary material for: Long COVID shows IL-1 pathway dysregulation without a discrete circulating biomarker signature
Source: Front Immunol. 2026 Oct 19;17:1957339. doi: 10.3389/fimmu.2026.1957339 (PMC13724367; doi:10.3389/fimmu.2026.1957339)
Supplement: Supplementary file 1 [file DataSheet1.pdf]

Supplementary Material for:

Long COVID shows IL-1 pathway dysregulation without a discrete circulating biomarker signature

Francisco Muñoz-López, Marissa Flores-Santamaría, Rebeca Santano, Macedonia Trigueros, Eva Riveira-Muñoz, Sara Cabrero de las Heras, Gooya Banaei, Edwards Pradenas, Benjamin Trinité, Silvia Marfil, Ana Gervassi, Víctor Urrea, Jon Izquierdo-Pujol, Cora Loste, José Ramón Santos, Gemma Lladós, Tomasso Francesco Aiello, Anna Chamorro, Alfons Jiménez, Carlota Dobaño, Roger Paredes, Julià Blanco, Ester Ballana, Gemma Moncunill, Lourdes Mateu, Maria Nevot, Marta Massanella

The PDF file includes:

- Tables S1 to S8
- Figures S1 to S8

Supplementary Table 1: Symptoms assessed in participants with LC. Symptoms were systematically assessed during face-to-face visits using a prespecified clinical questionnaire. A symptom was considered persistent when it was present at the study visit and had developed after, persisted from, or worsened following the acute SARS-CoV-2 infection. Related manifestations were grouped under the symptom categories indicated in the table.

	Symptoms	Specifications
Systemic	Fever	
	Low-grade fever	
	Temperature dysregulation	Chills; nocturnal chills; altered temperature sensation; hot flashes; shivering; sensation of abnormal body temperature; other.
	Diaphoresis	
	Fatigue	
	Weight loss	
	Loss of appetite	
	Reduced appetite	
Musculoskeletal	Arthralgia	
	Myalgia	
Pulmonary	Dyspnea	
	Cough	
	Sputum production	
Cardiovascular	Chest pain	
	Tachycardia	
	Blood pressure abnormalities	
Gastrointestinal	Dysphagia	Solids; liquids; both
	Diarrhea	
	Constipation	
	Nausea	
	Vomiting	
	Abdominal pain	
Neuropsychiatric	Neurocognitive complaints	Brain fog; impaired concentration; memory impairment; difficulty with planning; impaired attention; disorientation; anomia (word-finding difficulty); bradylalia (slowed speech); bradyphrenia (slowed thinking); difficulties with writing and/or reading.
	Headache	
	Neurosentive alterations	Paresthesia/dysesthesia; photophobia; phonophobia
	Dizziness	Vertigo; lightheadedness/dizziness; unsteadiness; other
	Insomnia	
HEENT	Smell alterations	
	Taste alterations	
	Otalgia	
	Dysphonia	
	Sore throat	
	Visual alterations	
	Tinnitus	
Dermatological	Mucosal dryness	
	Dermatological alterations	
	Hair loss	

**HEENT: Head, Eyes, Ears, Nose, and Throat.*

Supplementary Table 2. Demographic and clinical features of LC clusters of participants A, B, and C.

	Cluster A N = 53		Cluster B N = 38		Cluster C N = 48		P value	*Pairwise Holm-adjusted P-values*			*Absolute standardized mean differences*		
	Value	Missing	Value	Missing	Value	Missing	**Overall**	**A vs B**	**A vs C**	**B vs C**	**A vs B**	**A vs C**	**B vs C**
**Age,** years, median [IQR][1]	46 [38–55]	0	46 [37.2–52.5]	0	45 [38.8–53.2]	0	*0.964*	*1*	*1*	*1*	0.038	0.08	0.043
**Female sex**, N (%)[2]	32 (60.4)	0	25 (65.8)	0	42 (87.5)	0	*0.006*	*0.664*	*0.009*	*0.04*	0.112	0.65	0.531
**Time from symptoms onset to sample collection**, months, median [IQR][1]	8 [6.3–8.9]	0	8.2 [7.2–8.7]	0	8.5 [6.9–10]	0	*0.08*	*0.429*	*0.074*	*0.429*	0.198	0.48	0.3
**Number of persistent symptoms,** median [IQR][1]	5 [3–7]	0	10 [8.2–12]	0	17 [14–19]	0	*<0.001*	*<0.001*	*<0.001*	*<0.001*	1.724	3.645	1.972
**Hospitalization for acute COVID-19,** N (%)[2]	26 (49.1)	0	15 (39.5)	0	15 (31.2)	0	*0.189*	*0.799*	*0.312*	*0.799*	0.194	0.369	0.173
**ICU/RICU entry for acute COVID-19,** N (%)[2]	7 (13.2)	0	4 (10.5)	0	2 (4.2)	0	*0.286*	*0.797*	*0.494*	*0.797*	0.083	0.325	0.246
**COVID-19 Severity** , N (% of hospitalized)[2]							*0.749*						
High flow oxygen	8 (30.8)	0	4 (26.7)	0	3 (20.0)	0					0.091	0.249	0.158
Low flow oxygen	12 (46.2)	0	8 (53.3)	0	6 (40.0)	0					0.144	0.125	0.27
No oxygen required	4 (15.4)	0	3 (20.0)	0	5 (33.3)	0					0.121	0.428	0.305
**Diagnosis method, N (%)**[2]		0		0		0	*0.057*	*0.843*	*0.043*	*0.264*	0.114	0.59	0.469
Nasopharyngeal SARS-CoV-2 PCR	39 (73.6)		26 (68.4)		22 (45.8)								
Lateral Flow Rapid Test	0 (0.0)		0 (0.0)		1 (2.1)								
Serology	8 (15.1)		6 (15.8)		10 (20.8)								
Clinical	6 (11.3)		6 (15.8)		15 (31.2)								
**SARS-CoV-2 variant, N (%)**[2]		0		0		0	*0.559*	*1*	*1*	*1*	0.28	0.02	0.295
Pre-Alpha	51 (96.2)		38 (100.0)		46 (95.8)								
Alpha	2 (3.8)		0 (0.0)		2 (4.2)								
**Comorbidities,** N (%)[2]													
High blood pressure	13 (27.7)	6	6 (16.7)	2	8 (18.2)	4	*0.413*	*0.891*	*0.891*	*1*	0.267	0.227	0.04
Allergy	15 (31.9)	6	16 (44.4)	2	16 (36.4)	4	*0.484*	*0.785*	*0.997*	*0.997*	0.26	0.094	0.165
Diabetes	8 (17.0)	6	3 (8.3)	2	1 (2.3)	4	*0.045*	*0.643*	*0.093*	*0.643*	0.263	0.516	0.273
Obesity	13 (27.7)	6	11 (31.4)	3	14 (32.6)	5	*0.883*	*1*	*1*	*1*	0.083	0.107	0.024
Dyslipidemia	11 (23.4)	6	10 (27.8)	2	9 (20.5)	4	*0.753*	*1*	*1*	*1*	0.1	0.071	0.172
Ischemic heart disease	0 (0.0)	6	1 (2.8)	2	0 (0.0)	4	*0.283*	*0.867*		*0.867*	0.239	<0.001	0.239
Headache	5 (10.6)	6	3 (8.3)	2	6 (13.6)	4	*0.777*	*1*	*1*	*1*	0.079	0.092	0.17
Arrhythmia	1 (2.1)	6	5 (13.9)	2	2 (4.5)	4	*0.092*	*0.242*	*0.608*	*0.468*	0.444	0.135	0.327
Thromboembolic disease	1 (2.1)	6	0 (0.0)	2	0 (0.0)	4	*1*	*1*	*1*		0.209	0.209	<0.001
Stroke	0 (0.0)	6	0 (0.0)	2	0 (0.0)	4	-	-	-	-	<0.001	<0.001	<0.001
Heart failure	0 (0.0)	6	0 (0.0)	2	0 (0.0)	4	-	-	-	-	<0.001	<0.001	<0.001

[1] *Kruskal-Wallis test with pairwise Dunn's post-hoc comparisons;* [2] *Fisher's exact test*

Supplementary Table 3. Seropositivity rates for SARS-CoV-2-specific antibodies according to antigen and isotype.

	IgG				IgA				IgM			
	% Positive		P-value[1]	FDR P-value[2]	% Positive		P-value[1]	FDR P-value[2]	% Positive		P-value[1]	FDR P-value[2]
	LC	Recov.			LC	Recov.			LC	Recov.		
**RBD**	61.9	73.7	*0.09*	*0.22*	64.7	72.7	*0.21*	*0.32*	31.7	33.3	*0.89*	*1.00*
**S**	69.1	77.8	*0.14*	*0.25*	64.7	74.7	*0.12*	*0.25*	9.4	8.1	*0.82*	*1.00*
**S1**	69.9	82.8	***0.03***	*0.10*	66.9	77.8	*0.08*	*0.22*	32.4	32.3	*1.00*	*1.00*
**S2**	77	88.9	***0.03***	*0.10*	77	88.9	***0.03***	*0.10*	12.2	11.11	*0.84*	*1.00*
**N**	22.3	31.3	*0.14*	*0.25*	20.2	23.23	*0.63*	*0.92*	1.4	1.01	*1.00*	*1.00*
**Any positive**	77.7	90.1	***0.008***	*0.09*	78.4	88.9	***0.04***	*0.12*	39.6	40.4	*1.00*	*1.00*

[1] P-values were calculated using Fisher's exact tests and [2] corrected with FDR method

Supplementary Table 4. Demographic and clinical characteristics of the study population included in the sensitivity analysis restricted to laboratory-confirmed SARS-CoV-2 infection.

	Uninfected N = 74		Recovered N=96		LC N =112		*P-value*	*Pairwise Holm-adjusted P-values*			Absolute standardized mean differenc		
	Value	Missing	Value	Missing	Value	Missing	***Overall***	**U vs R**	**U vs LC**	**R vs LC**	**U vs R**	**U vs LC**	**R vs LC**
**Age,** years, median [IQR][1]	42 [32.2–52.8]	0	53 [41.8–62]	0	47 [40–55]	0	***<0.001***	***<0.001***	*0.07*	*0.07*	0.569	0.325	0.263
**Female Sex**, N (%)[2]	47 (63.5%)	0	40 (41.7%)	0	78 (69.6%)	0	***<0.001***	***0.011***	*0.427*	***<0.001***	0.448	0.13	0.587
**Time from symptoms onset to sample collection**, months, median [IQR	–	–	7.2 [6.4–8.3]	0	8 [6.6–8.8]	0	***0.05***			***0.05***			0.221
**Hospitalization for acute COVID-19**, N (%)[2]	–	–	52 (54.2%)	0	53 (47.3%)	0	*0.334*			*0.334*			0.137
**ICU/RICU entry for acute COVID-19**, N (%)[2]	–	–	7 (7.3%)	0	13 (11.6%)	0	*0.35*			*0.35*			0.148
**COVID-19 Severity** , N (% of hospitalized)[2]													
High-flow oxygen	–	–	9 (17.3%)	0	14 (26.4%)	0	*0.346*			*0.346*			0.222
Low-flow oxygen	–	–	26 (50.0%)	0	26 (49.1%)	0	*1*			*1*			0.019
No oxygen requirement	–	–	14 (26.9%)	0	10 (18.9%)	0	*0.36*			*0.36*			0.193
**Diagnosis method**, N (%)[2]	–			0		0	*0.356*			*0.356*			0.157
Nasopharyngeal SARS-CoV-2 PCR	–	–	68 (70.8%)	0	87 (77.7%)	0							
Lateral Flow Rapid Test	–	–	3 (3.1%)	0	1 (0.9%)	0							
Serology	–	–	25 (26.0%)	0	24 (21.4%)	0							
**SARS-CoV-2 Vaccination before sampling**, N (%)[2]	0 (0.0%)	0	0 (0.0%)	0	0 (0.0%)	0					0	0	0
**SARS-CoV-2 variant**, N (%)[2]	–			0		0	*1*			*1*			0.025
Pre-Alpha	–	–	93 (96.9%)	0	108 (96.4%)	0							
Alpha	–	–	3 (3.1%)	0	4 (3.6%)	0							
**Comorbidities,** N (%)[2]													
High blood pressure	4 (14.8%)	47	25 (35.2%)	25	27 (26.2%)	9	*0.121*	*0.157*	*0.478*	*0.478*	0.485	0.285	0.196
Allergy	3 (11.1%)	47	19 (26.4%)	24	36 (35.0%)	9	***0.04***	*0.346*	*0.054*	*0.346*	0.399	0.59	0.186
Diabetes	1 (3.7%)	47	13 (18.1%)	24	12 (11.7%)	9	*0.156*	*0.311*	*0.55*	*0.55*	0.474	0.302	0.181
Obesity	1 (3.8%)	48	14 (19.7%)	25	34 (33.7%)	11	***0.002***	*0.115*	***0.004***	*0.115*	0.508	0.827	0.319
Dyslipidemia	7 (25.9%)	47	30 (41.7%)	24	26 (25.2%)	9	*0.064*	*0.339*	*1*	*0.095*	0.337	0.016	0.353
Ischemic heart disease	2 (7.4%)	47	6 (8.3%)	24	1 (1.0%)	9	***0.032***	*1*	*0.218*	*0.06*	0.034	0.326	0.355
Headache	2 (7.4%)	47	5 (6.9%)	24	12 (11.7%)	9	*0.598*	*1*	*1*	*1*	0.018	0.145	0.163
Arrhythmia	2 (7.4%)	47	6 (8.3%)	24	6 (5.8%)	9	*0.738*	*1*	*1*	*1*	0.034	0.064	0.098
Thromboembolic disease	1 (3.7%)	47	1 (1.4%)	24	1 (1.0%)	9	*0.526*	*1*	*1*	*1*	0.147	0.182	0.039
Stroke	0 (0.0%)	47	1 (1.4%)	24	0 (0.0%)	9	*0.49*	*1*		*0.823*	0.168	0	0.168
Heart failure	0 (0.0%)	47	1 (1.4%)	24	0 (0.0%)	9	*0.49*	*1*		*0.823*	0.168	0	0.168

[1] Kruskal-Wallis test with pairwise Dunn's post-hoc comparisons; [2] Fisher's exact test; [3] Wilcoxon rank-sum test

Supplementary Table 5: Cross-validated performance of random forest classifiers across classification tasks and biomarker panels

A. Overall model performance using all available participants

Classification endpoint	Predictor panel	N	Class distribution, n	Predictors, n	Accuracy (95% CI)	Balanced accuracy (95% CI)	Sensitivity (95% CI)	Specificity (95% CI)	ROC AUC (95% CI)
Three-group classification	Immune biomarkers	312	Uninf, N=74; Recov., N=99; LC, N= 139	23	0.506 (0.449–0.558)	0.639 (0.596–0.677)	0.522 (0.464–0.573)	0.756 (0.728–0.782)	0.712 (0.670–0.750)
	Immune and endothelial biomarkers	312	Uninf, N=74; Recov., N=99; LC, N= 139	28	0.542 (0.484–0.596)	0.666 (0.625–0.704)	0.560 (0.506–0.611)	0.771 (0.743–0.798)	0.750 (0.709–0.789)
Infection status	Immune biomarkers	312	Uninf., N=74; Infected, N=238	23	0.663 (0.612–0.715)	0.719 (0.667–0.772)	0.613 (0.554–0.675)	0.824 (0.735–0.909)	0.781 (0.722–0.837)
	Immune and endothelial biomarkers	312	Uninf., N=74; Infected, N=238	28	0.702 (0.651–0.750)	0.735 (0.676–0.788)	0.672 (0.611–0.731)	0.797 (0.703–0.886)	0.820 (0.759–0.876)
Long COVID status	Luminex + TGFb	238	Recov., N=99; LC, 139	23	0.601 (0.542–0.664)	0.602 (0.541–0.662)	0.597 (0.518–0.677)	0.606 (0.512–0.704)	0.634 (0.562–0.704)
	Luminex + endothelial	238	Recov., N=99; LC, 139	28	0.622 (0.563–0.685)	0.627 (0.567–0.691)	0.597 (0.514–0.677)	0.657 (0.569–0.750)	0.645 (0.575–0.715)
	Integrated multimodal	220	Recov., 85; LC, 135	60	0.605 (0.536–0.668)	0.617 (0.549–0.682)	0.563 (0.476–0.645)	0.671 (0.566–0.769)	0.640 (0.561–0.711)

B. Class-specific performance for the three-group classification

Predictor panel	Class	Class n	Sensitivity	Specificity	Balanced accuracy
Immune biomarkers	Uninfected	74	0.689	0.718	0.704
	Recovered	99	0.374	0.775	0.574
	Long COVID	139	0.504	0.775	0.639
Immune and endothelial biomarkers	Uninfected	74	0.757	0.756	0.757
	Recovered	99	0.384	0.789	0.586
	Long COVID	139	0.540	0.769	0.654

C. Performance of predictor panels for Long COVID versus recovered participants in the common complete-case cohort

Predictor panel	N	Class distribution, n	Predictors, n	Accuracy (95% CI)	Balanced accuracy (95% CI)	Sensitivity (95% CI)	Specificity (95% CI)	ROC AUC (95% CI)
Immune biomarkers	220	Recov., N=85; LC, N=135	23	0.582 (0.518–0.645)	0.581 (0.515–0.649)	0.585 (0.500–0.667)	0.576 (0.474–0.681)	0.599 (0.519–0.677)
Immune and endothelial biomarkers	220	Recov., N=85; LC, N=135	28	0.573 (0.509–0.641)	0.576 (0.511–0.645)	0.563 (0.480–0.645)	0.588 (0.481–0.697)	0.604 (0.528–0.683)
Integrated multimodal	220	Recov., N=85; LC, N=135	60	0.609 (0.541–0.673)	0.620 (0.555–0.683)	0.570 (0.486–0.652)	0.671 (0.575–0.768)	0.633 (0.558–0.707)

D. Paired differences in performance between predictor panels in the common complete-case cohort

Comparison (first vs. second)	Δ Accuracy (95% CI)	Δ Balanced accuracy (95% CI)	Δ Sensitivity (95% CI)	Δ Specificity (95% CI)	Δ ROC AUC (95% CI)
Immune and endothelial biomarkers vs. Immune biomarkers	−0.009 (−0.055–0.041)	−0.005 (−0.054–0.047)	−0.022 (−0.081–0.038)	0.012 (−0.069–0.100)	0.005 (−0.030–0.042)
Integrated multimodal vs. Immune and endothelial biomarkers	0.036 (−0.036–0.109)	0.045 (−0.031–0.118)	0.007 (−0.077–0.092)	0.082 (−0.034–0.202)	0.029 (−0.038–0.096)
Integrated multimodal vs. Immune biomarkers	0.027 (−0.045–0.105)	0.040 (−0.040–0.123)	−0.015 (−0.103–0.071)	0.094 (−0.042–0.235)	0.035 (−0.039–0.107)

Values are consensus out-of-fold estimates with participant-level bootstrap 95% CIs unless otherwise indicated. Models used stratified five-fold cross-validation repeated 20 times with 500 trees; mtry was tuned within each training fold using out-of-bag balanced accuracy. For binary classifications, sensitivity refers to Infected or Long COVID; for the three-group classification, performance metrics in panel A are macro-averaged across classes. Class-specific estimates in panel B are reported without CIs. Panels A–B use the maximum complete-case sample available for each predictor panel. Panels C–D compare all three predictor panels in the same 220 participants (Recovered, n=85; Long COVID, n=135) using identical outer cross-validation folds. Paired differences in panel D were estimated using participant-level bootstrap resampling (2,000 iterations); positive values favor the first panel named. All paired 95% CIs included zero. Random forest analyses were exploratory and were not developed or externally validated as diagnostic models. ROC AUC, area under the receiver operating characteristic curve; CI, confidence interval; Δ, difference.

Supplementary Table 6. Eligibility, performance, and predictor stability of penalized logistic regression models for persistent symptoms among PWLC. Models were evaluated in the predictor-complete analytical dataset ($N = 135$). Symptoms were eligible for modeling only when at least 20 participants were symptom-present and at least 20 were symptom-absent; excluded symptoms and the corresponding reason are shown. Elastic net models ($\alpha = 0.5$) were evaluated using repeated nested stratified 5-fold cross-validation, with model tuning performed within each training set. Performance metrics were calculated from out-of-fold (OOF) predictions. ROC AUC is reported with 95% bootstrap confidence intervals; AUPRC baseline corresponds to symptom prevalence. Balanced accuracy is the mean of sensitivity and specificity. The final column lists predictors recurrently selected across resamples; the sign indicates the direction of the penalized coefficient, and the percentage indicates selection frequency.

Symptom	N	Present, n (%)	Absent, n (%)	Eligibility	Exclusion reason	OOF ROC AUC (95% CI)	AUPRC	AUPRC baseline (prevalence)	Balanced accuracy	Sensitivity	Specificity	Recurrently selected predictors (75/90)
Fatigue	135	114 (84.4%)	21 (15.6%)	Eligible		0.70 (0.59–0.81)	0.93	0.844	0.496	0.991	0	ANG-1 (+; 84%); IgG CMV pp150 (+; 100%)
Low-grade fever	135	32 (23.7%)	103 (76.3%)	Eligible		0.69 (0.59–0.79)	0.4	0.237	0.5	0	1	IgG SARS-CoV-2 S2 (−; 97%); IgG SARS-CoV-2 S (−; 75%); Age (−; 96%)
Myalgia	135	62 (45.9%)	73 (54.1%)	Eligible		0.60 (0.50–0.69)	0.572	0.459	0.565	0.5	0.63	IL-1RA (+; 87%); IL-13 (−; 82%); EGF (+; 79%); TGF-β (−; 98%); ET-1 (−; 81%); IgA HSV-1 (−; 98%); IgA EBV ZEBRA (−; 81%); IgG EBV VCAp18 (−; 79%); IgG CMV pp150 (+; 77%)
Headache	135	77 (57.0%)	58 (43.0%)	Eligible		0.60 (0.50–0.69)	0.65	0.57	0.582	0.818	0.345	IL-1RA (+; 99%)
Abdominal pain	135	35 (25.9%)	100 (74.1%)	Eligible		0.60 (0.50–0.70)	0.314	0.259	0.511	0.143	0.88	IL-6 (−; 94%); IL-8 (+; 92%); Eotaxin (−; 84%); IL-2 (−; 83%); P-selectin (−; 99%); ET-1 (−; 97%); ANG-2 (−; 91%); IgM SARS-CoV-2 S1 (−; 88%); IgM SARS-CoV-2 RBD (−; 80%); IgA EBV VCAp18 (−; 90%); IgA EBV EAD (+; 85%); IgA VZV gE (+; 85%); IgG HSV-1 (+; 82%); IgA EBV ZEBRA (−; 77%); IgA HHV-6A (+; 75%)
Neurocognitive alterations	135	95 (70.4%)	40 (29.6%)	Eligible		0.60 (0.48–0.71)	0.753	0.704	0.559	0.842	0.275	GM-CSF (−; 91%); IP-10 (+; 79%); ANG-1 (+; 97%); IgA SARS-CoV-2 S (−; 85%); IgA SARS-CoV-2 RBD (−; 81%); IgG HHV-6A (+; 90%); Age (+; 76%)
Hair loss	135	61 (45.2%)	74 (54.8%)	Eligible		0.59 (0.50–0.69)	0.544	0.452	0.529	0.328	0.73	IL-1RA (+; 98%)
Taste alterations	135	39 (28.9%)	96 (71.1%)	Eligible		0.59 (0.48–0.69)	0.361	0.289	0.537	0.231	0.844	IL-8 (+; 86%); IL-13 (−; 80%); IL-2R (+; 78%); IL-7 (+; 77%); TGF-β (−; 81%); sEPCR (+; 80%); IgA SARS-CoV-2 S2 (+; 84%); IgA SARS-CoV-2 S (+; 78%); IgA EBV ZEBRA (−; 97%); IgM EBV VCAp18 (+; 77%); IgG EBV VCAp18 (+; 76%); IgM EBV EAD (−; 76%); Male sex (Female reference) (−; 79%); Age (−; 77%)
Tachycardia	135	55 (40.7%)	80 (59.3%)	Eligible		0.58 (0.48–0.67)	0.482	0.407	0.553	0.382	0.725	RANTES (+; 98%); G-CSF (−; 82%); IL-10 (+; 79%); IgM SARS-CoV-2 S (−; 88%); IgG SARS-CoV-2 S2 (−; 78%); IgA CMV pp150 (−; 76%); Male sex (Female reference) (−; 86%)
Dysphonia	135	33 (24.4%)	102 (75.6%)	Eligible		0.57 (0.45–0.68)	0.301	0.244	0.528	0.212	0.843	IL-13 (−; 84%); IL-8 (+; 81%); IL-10 (−; 77%); TNF-α (+; 75%); TGF-β (−; 84%); ANG-2 (−; 78%); IgA SARS-CoV-2 S (−; 88%); IgM SARS-CoV-2 S (−; 85%); IgM HHV-6A (−; 93%); IgM EBV EAD (−; 86%); IgG HHV-6A (−; 83%); IgG HSV-1 (+; 82%); IgG EBV ZEBRA (−; 77%); Male sex (Female reference) (−; 79%)
Chest pain	135	73 (54.1%)	62 (45.9%)	Eligible		0.56 (0.46–0.66)	0.565	0.541	0.552	0.685	0.419	IL-8 (+; 87%); IL-1RA (+; 86%); ET-1 (−; 87%); IgM EBV ZEBRA (+; 76%); IgG HHV-6A (+; 75%)
Diarrhea	135	50 (37.0%)	85 (63.0%)	Eligible		0.54 (0.44–0.64)	0.455	0.37	0.541	0.2	0.882	IgG EBV EAD (+; 96%)
Mucosal dryness	135	39 (28.9%)	96 (71.1%)	Eligible		0.54 (0.43–0.65)	0.316	0.289	0.501	0.179	0.823	IFN-γ (−; 84%); TGF-β (−; 89%); ET-1 (−; 83%); ANG-1 (+; 81%); IgA SARS-CoV-2 N (−; 85%); IgA SARS-CoV-2 S (−; 81%); IgG EBV ZEBRA (−; 97%); IgA HHV-6A (−; 89%); IgA EBV EAD (+; 86%); IgG HSV-1 (+; 81%); IgG HHV-6A (+; 77%); Male sex (Female reference) (−; 76%)
Temperature alterations	135	29 (21.5%)	106 (78.5%)	Eligible		0.54 (0.42–0.66)	0.237	0.215	0.478	0.069	0.887	P-selectin (−; 87%); IgA SARS-CoV-2 S (−; 82%); IgA EBV EBNA (+; 79%); IgG EBV VCAp18 (−; 76%); Age (−; 77%)
Tinnitus	135	28 (20.7%)	107 (79.3%)	Eligible		0.52 (0.39–0.63)	0.266	0.207	0.509	0.036	0.981	ANG-2 (−; 89%)
Neurosensory alterations	135	51 (37.8%)	84 (62.2%)	Eligible		0.52 (0.42–0.62)	0.387	0.378	0.488	0.275	0.702	TNF-α (−; 88%); IL-12/IL-23p40 (+; 85%); P-selectin (−; 81%); TGF-β (−; 76%); IgA SARS-CoV-2 N (−; 93%); IgM SARS-CoV-2 S2 (−; 77%); IgA HHV-6A (−; 94%); IgG CMV pp150 (+; 80%); IgA HSV-1 (−; 77%)
Arthralgia	135	83 (61.5%)	52 (38.5%)	Eligible		0.51 (0.41–0.62)	0.605	0.615	0.53	0.867	0.192	IL-1RA (+; 87%); ET-1 (−; 90%)
Smell alterations	135	49 (36.3%)	86 (63.7%)	Eligible		0.50 (0.40–0.60)	0.399	0.363	0.5	0.163	0.837	IL-8 (+; 95%)
Visual alterations	135	29 (21.5%)	106 (78.5%)	Eligible		0.50 (0.38–0.63)	0.23	0.215	0.48	0.034	0.925	IgG EBV ZEBRA (−; 93%); IgG EBV VCAp18 (−; 77%)
Insomnia	135	31 (23.0%)	104 (77.0%)	Eligible		0.49 (0.35–0.62)	0.273	0.23	0.523	0.161	0.885	Eotaxin (−; 77%); IL-1RA (+; 77%); IgA EBV EBNA (−; 86%); IgG EBV EBNA (−; 76%)
Dyspnea	135	89 (65.9%)	46 (34.1%)	Eligible		0.46 (0.36–0.57)	0.61	0.659	0.504	0.899	0.109	IgA HSV-1 (−; 82%); IgG HHV-6A (+; 81%); IgG CMV pp150 (+; 77%)
Instability	135	31 (23.0%)	104 (77.0%)	Eligible		0.46 (0.36–0.56)	0.204	0.23	0.5	0	1,000	IL-8 (+; 91%); IL-1RA (+; 77%)
Cough	135	46 (34.1%)	89 (65.9%)	Eligible		0.42 (0.32–0.53)	0.321	0.341	0.487	0.109	0.865	ANG-2 (−; 79%); IgA SARS-CoV-2 S1 (−; 78%)
Skin alterations	135	51 (37.8%)	84 (62.2%)	Eligible		0.41 (0.32–0.51)	0.328	0.378	0.418	0.157	0.679	P-selectin (−; 76%); IgG CMV pp150 (+; 80%)
Constipation	135	22 (16.3%)	113 (83.7%)	Eligible		0.39 (0.26–0.52)	0.136	0.163	0.5	0	1	None
Fever	135	7 (5.2%)	128 (94.8%)	Excluded	<20 events/non-events							
Diaphoresis	135	8 (5.9%)	127 (94.1%)	Excluded	<20 events/non-events							
Expectoration	135	15 (11.1%)	120 (88.9%)	Excluded	<20 events/non-events							
Otalgia	135	6 (4.4%)	129 (95.6%)	Excluded	<20 events/non-events							
Nausea	135	16 (11.9%)	119 (88.1%)	Excluded	<20 events/non-events							
Odynophagia	135	19 (14.1%)	116 (85.9%)	Excluded	<20 events/non-events							
Weight loss	135	7 (5.2%)	128 (94.8%)	Excluded	<20 events/non-events							
Anorexia	135	6 (4.4%)	129 (95.6%)	Excluded	<20 events/non-events							
Hyporexia	135	5 (3.7%)	130 (96.3%)	Excluded	<20 events/non-events							
Blood pressure alterations	135	13 (9.6%)	122 (90.4%)	Excluded	<20 events/non-events							
Dysphagia	135	18 (13.3%)	117 (86.7%)	Excluded	<20 events/non-events							

All 36 symptom outcomes are shown. Models were fitted only for symptoms with at least 20 participants in both outcome classes. Recurrent predictors are summarized using the $\geq$75% selection-frequency and $\geq$90% sign-consistency visualization criterion.

Supplementary Table 7. Target-specific positive RT-qPCR wells among RNA-positive samples.

**Target**	**Positive wells per sample**	**Recovered N = 4**	**Long COVID N = 10**	**Total N = 14**
**Any target**, (n%)	At least 2 positive wells	3 (75.0)	6 (60.0)	9 (64.3)
**N and E**, n (%)	Both genomic targets detected	0 (0.0)	1 (10.0)	1 (7.1)
**N target**, n (%)				
	0	0 (0.0)	1 (10.0)	1 (7.1)
	1	1 (25.0)	3 (30.0)	4 (28.6)
	2	1 (25.0)	0 (0.0)	1 (7.1)
	4	2 (50.0)	4 (40.0)	6 (42.9)
	8	0 (0.0)	2 (20.0)	2 (14.3)
**E target**, n (%)				
	0	4 (100.0)	6 (60.0)	10 (71.4)
	1	0 (0.0)	2 (20.0)	2 (14.3)
	Not assessed	0 (0.0)	2 (20.0)	2 (14.3)

A sample was considered RNA-positive when at least one second-round RT-qPCR well was positive for the N or E target. A negative sample required zero positive wells for both targets. E-target results were unavailable for two long COVID RNA-positive samples.

Supplementary Table 8. Multivariable-adjusted comparisons of biomarkers across long COVID symptom clusters. Primary models were adjusted for age, sex and hospitalization during acute COVID-19.The sensitivity model additionally included obesity and was restricted to participants with available obesity data.

Biomarker	n	Primary model adjusted p-value	A − B β (95% CI)	Adjusted p-value	A − C β (95% CI)	Adjusted p-value	B − C β (95% CI)	Adjusted p-value	n with obesity data	Obesity-sensitivity adjusted p-value
**Soluble immune biomarkers**										
IL-1b	139	*0.487*	0.111 (-0.062 to 0.285)	*0.415*	-0.027 (-0.196 to 0.141)	*0.957*	-0.138 (-0.318 to 0.042)	*0.378*	125	*0.31*
G-CSF	139	*0.423*	0.105 (-0.010 to 0.221)	*0.356*	0.009 (-0.103 to 0.122)	*0.957*	-0.096 (-0.216 to 0.024)	*0.378*	125	*0.292*
IL-10	139	*0.487*	0.134 (-0.095 to 0.363)	*0.458*	-0.037 (-0.259 to 0.185)	*0.957*	-0.171 (-0.408 to 0.066)	*0.378*	125	*0.621*
IL-13	139	*0.651*	0.070 (-0.082 to 0.222)	*0.629*	-0.022 (-0.170 to 0.125)	*0.957*	-0.092 (-0.250 to 0.066)	*0.378*	125	*0.642*
IL-6	139	*0.423*	0.304 (0.055 to 0.554)	*0.295*	0.137 (-0.105 to 0.379)	*0.91*	-0.168 (-0.426 to 0.091)	*0.378*	125	*0.292*
IL12-IL23p40	139	*0.446*	0.072 (-0.036 to 0.181)	*0.415*	-0.027 (-0.132 to 0.079)	*0.957*	-0.099 (-0.212 to 0.014)	*0.338*	125	*0.292*
RANTES	139	*0.828*	-0.004 (-0.039 to 0.031)	*0.965*	-0.011 (-0.045 to 0.023)	*0.957*	-0.007 (-0.043 to 0.029)	*0.767*	125	*0.569*
EOTAXIN	139	*0.711*	-0.008 (-0.118 to 0.102)	*0.966*	0.045 (-0.061 to 0.152)	*0.957*	0.053 (-0.061 to 0.167)	*0.429*	125	*0.958*
GM-CSF	139	*0.711*	0.055 (-0.201 to 0.312)	*0.893*	-0.075 (-0.324 to 0.174)	*0.957*	-0.130 (-0.397 to 0.136)	*0.429*	125	*0.765*
MIP-1b	139	*0.423*	0.183 (0.010 to 0.356)	*0.307*	0.005 (-0.163 to 0.173)	*0.957*	-0.179 (-0.358 to 0.001)	*0.338*	125	*0.292*
MCP-1	139	*0.487*	0.098 (-0.040 to 0.237)	*0.415*	0.015 (-0.119 to 0.149)	*0.957*	-0.083 (-0.227 to 0.060)	*0.378*	125	*0.292*
EGF	139	*0.423*	0.144 (-0.028 to 0.316)	*0.4*	-0.018 (-0.185 to 0.149)	*0.957*	-0.162 (-0.341 to 0.017)	*0.338*	125	*0.292*
HGF	139	*0.423*	0.036 (-0.060 to 0.133)	*0.734*	-0.064 (-0.158 to 0.030)	*0.737*	-0.101 (-0.201 to 0.000)	*0.338*	125	*0.292*
IFNg	139	*0.828*	0.049 (-0.098 to 0.196)	*0.77*	0.009 (-0.134 to 0.152)	*0.957*	-0.040 (-0.192 to 0.113)	*0.694*	125	*0.765*
IFNa	139	*0.487*	0.124 (-0.051 to 0.299)	*0.415*	0.008 (-0.162 to 0.178)	*0.957*	-0.116 (-0.298 to 0.066)	*0.378*	125	*0.292*
IL-1RA	139	*0.423*	0.020 (-0.180 to 0.220)	*0.965*	-0.174 (-0.368 to 0.021)	*0.737*	-0.194 (-0.401 to 0.014)	*0.338*	125	*0.292*
TNFa	139	*0.423*	0.150 (0.020 to 0.281)	*0.295*	0.086 (-0.041 to 0.213)	*0.737*	-0.065 (-0.200 to 0.071)	*0.429*	125	*0.292*
IL-2	139	*0.423*	0.233 (-0.004 to 0.471)	*0.322*	0.059 (-0.172 to 0.289)	*0.957*	-0.175 (-0.421 to 0.071)	*0.378*	125	*0.292*
IL-7	139	*0.673*	0.003 (-0.156 to 0.161)	*0.994*	-0.077 (-0.231 to 0.077)	*0.957*	-0.080 (-0.244 to 0.085)	*0.429*	125	*0.678*
IP10	139	*0.828*	0.026 (-0.066 to 0.118)	*0.821*	0.022 (-0.067 to 0.111)	*0.957*	-0.004 (-0.099 to 0.092)	*0.94*	125	*0.621*
IL-2R	139	*0.487*	0.127 (-0.054 to 0.308)	*0.415*	0.018 (-0.158 to 0.193)	*0.957*	-0.109 (-0.297 to 0.078)	*0.378*	125	*0.457*
IL-8	139	*0.487*	-0.001 (-0.175 to 0.174)	*0.994*	-0.123 (-0.293 to 0.046)	*0.737*	-0.122 (-0.303 to 0.059)	*0.378*	125	*0.292*
TGFb	139	*0.423*	-0.023 (-0.196 to 0.151)	*0.965*	0.136 (-0.032 to 0.304)	*0.737*	0.159 (-0.021 to 0.338)	*0.338*	125	*0.569*
Ratio IL-1b/I-L1RA	139	*0.446*	0.140 (-0.070 to 0.350)	*0.415*	0.176 (-0.028 to 0.380)	*0.737*	0.036 (-0.182 to 0.254)	*0.777*	125	*0.292*
**Endothelial biomarkers**										
P-Selectin	139	*0.327*	-0.033 (-0.101 to 0.035)	*0.562*	0.050 (-0.016 to 0.116)	*0.342*	0.083 (0.013 to 0.154)	*0.107*	125	*0.148*
ANG-1	139	*0.543*	-0.079 (-0.193 to 0.034)	*0.562*	-0.088 (-0.198 to 0.022)	*0.342*	-0.009 (-0.127 to 0.109)	*0.88*	125	*0.198*
ANG-2	139	*0.706*	0.036 (-0.036 to 0.108)	*0.562*	0.029 (-0.041 to 0.099)	*0.521*	-0.007 (-0.082 to 0.068)	*0.88*	125	*0.573*
ET-1	139	*0.546*	-0.012 (-0.078 to 0.055)	*0.774*	0.037 (-0.027 to 0.102)	*0.419*	0.049 (-0.020 to 0.118)	*0.401*	125	*0.198*
EPCR	139	*0.852*	0.022 (-0.126 to 0.170)	*0.774*	-0.022 (-0.166 to 0.121)	*0.759*	-0.044 (-0.197 to 0.110)	*0.88*	125	*0.573*

Adjusted p-values are Benjamini–Hochberg FDR-adjusted within each biomarker family and model. β values are differences in log10-transformed biomarker concentrations; positive values indicate a higher estimated value in the first-named cluster.

Supplementary Figure 1

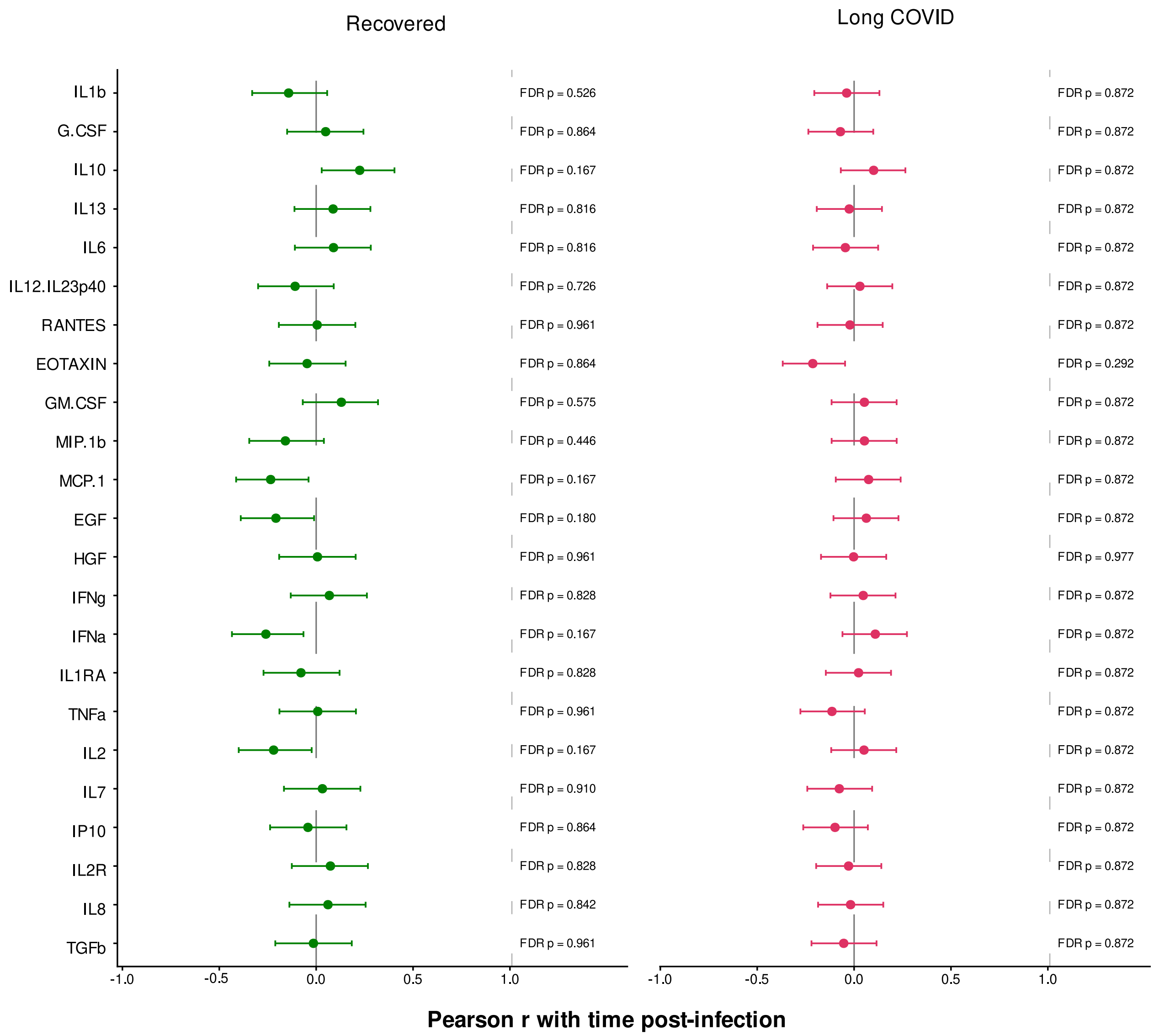

Supplementary Figure 1. Association between time post-infection and circulating biomarker levels. Forest plots show Pearson correlation coefficients (r) and 95% confidence intervals for the association between time since SARS-CoV-2 infection and circulating biomarker levels in recovered participants (left) and PWLC (right). Biomarker concentrations were normalized and log10-transformed before analysis. The vertical dashed line indicates r = 0 (no correlation). Correlations were evaluated separately for each biomarker, and P-values were adjusted for multiple testing using the FDR method.

Supplementary Figure 2

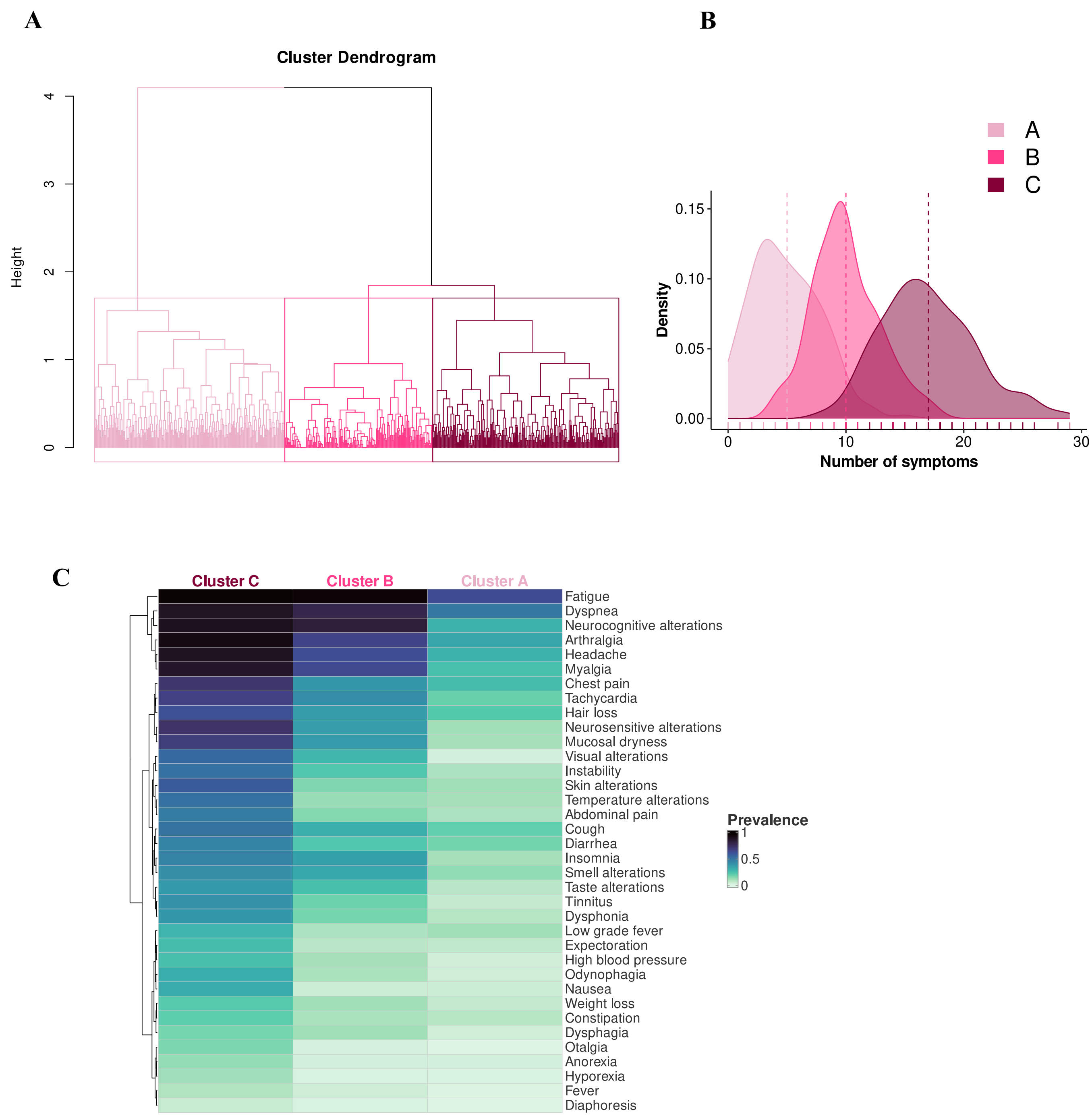

Supplementary Figure 2. Symptom-based clustering of PWLC. A) Hierarchical clustering dendrogram of 643 PWLC from the KING Cohort Extension based on symptom profiles using Gower distance. The optimal number of clusters was evaluated by silhouette width analysis. **B**) Density distribution of the number of reported simultaneous symptoms across clusters A, B, and C. Vertical dashed lines indicate median values for each cluster. **C**) Heatmap showing the prevalence of individual symptoms across the three clusters. Color intensity represents symptom prevalence within each cluster.

Supplementary Figure 3

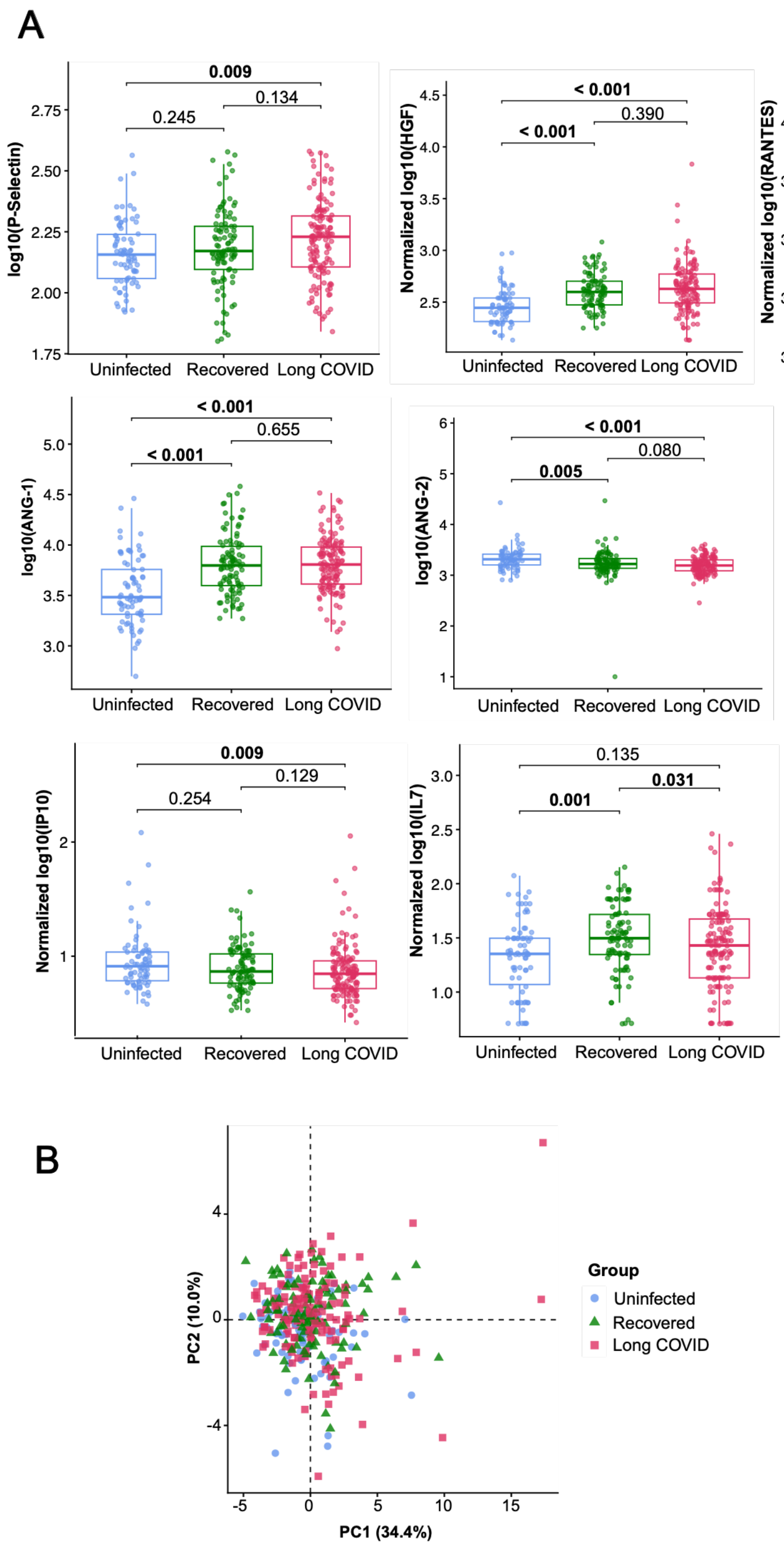

Supplementary Figure 3. **Cytokines, chemokines, and endothelial markers**. **A**) Normalized log10-transformed concentrations of soluble biomarkers measured in uninfected controls, recovered individuals, and PWLC. Statistical comparisons between groups were performed using Kruskal–Wallis tests followed by Dunn's post-hoc tests with Holm correction. Center lines represent medians, boxes indicate interquartile ranges, and whiskers represent 1.5× the interquartile range. **B**) PCA of normalized soluble biomarker profiles. Each point represents one participant, colored according to study group. Percentage of variance explained by each principal component is indicated on the axes.

Supplementary Figure 4

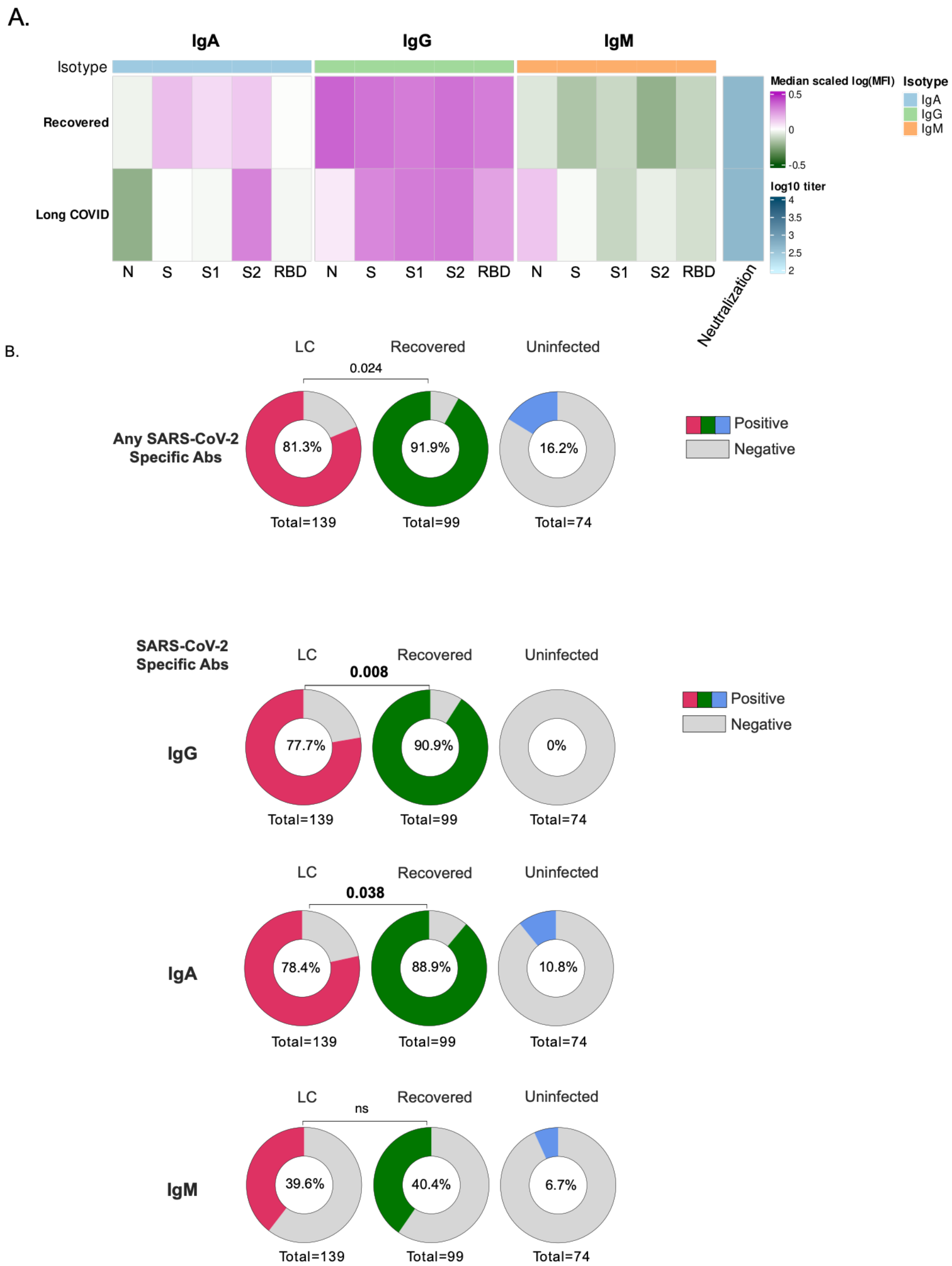

Supplementary Figure 4. SARS-CoV-2-specific humoral responses. A. Heatmap showing median scaled MFI values of anti-SARS-CoV-2 IgG, IgA, and IgM antibodies against nucleocapsid (N), spike (S), spike subunit 1 (S1), spike subunit 2 (S2), and receptor-binding domain (RBD) antigens in recovered individuals and PWLC. Neutralizing antibody titers against WH1 SARS-CoV-2 are shown on the right. Color intensity represents scaled median antibody levels or neutralization titers for each group and antigen–isotype combination. **B**. Frequency of participants positive for any SARS-CoV-2-specific antibodies (defined as positivity for IgG, IgA, or/and IgM; upper panel) and for each individual antibody isotype (IgG, IgA, and IgM; lower panels) against N, S, S1, S2, and RBD antigens in PWLC, recovered and uninfected. Statistical comparisons were performed between PWLC and recovered individuals using Fisher's exact test. The uninfected group is shown as a reference for background seroreactivity and was not included in the statistical comparisons.

Supplementary Figure 5

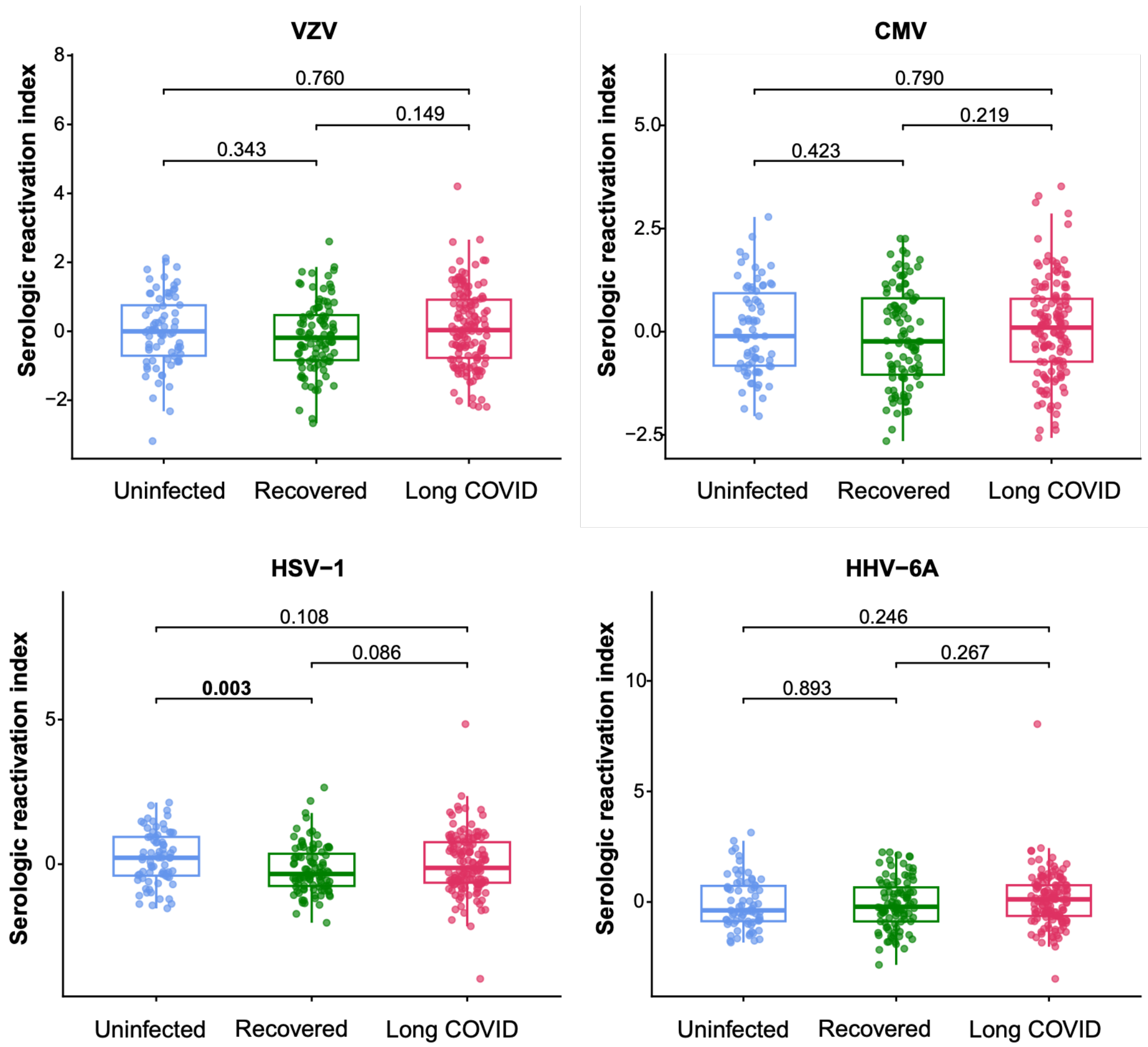

Supplementary Figure 5. Reactivation index of VZV, CMV, HSV1, and HHV6A. Reactivation indices for varicella-zoster virus (VZV), cytomegalovirus (CMV), herpes simplex virus type 1 (HSV1), and human herpesvirus 6A (HHV6A) in uninfected controls, recovered individuals and PWLC. Reactivation indices were calculated from normalized antibody responses associated with viral reactivation and latency, as described in the Supplementary Methods. Statistical comparisons between groups were performed using Kruskal–Wallis tests followed by Dunn's post-hoc tests with Holm correction. Exact adjusted p-values are indicated in the figure. Center lines represent medians, boxes indicate interquartile ranges, and whiskers represent 1.5× the interquartile range.

Supplementary Figure 6

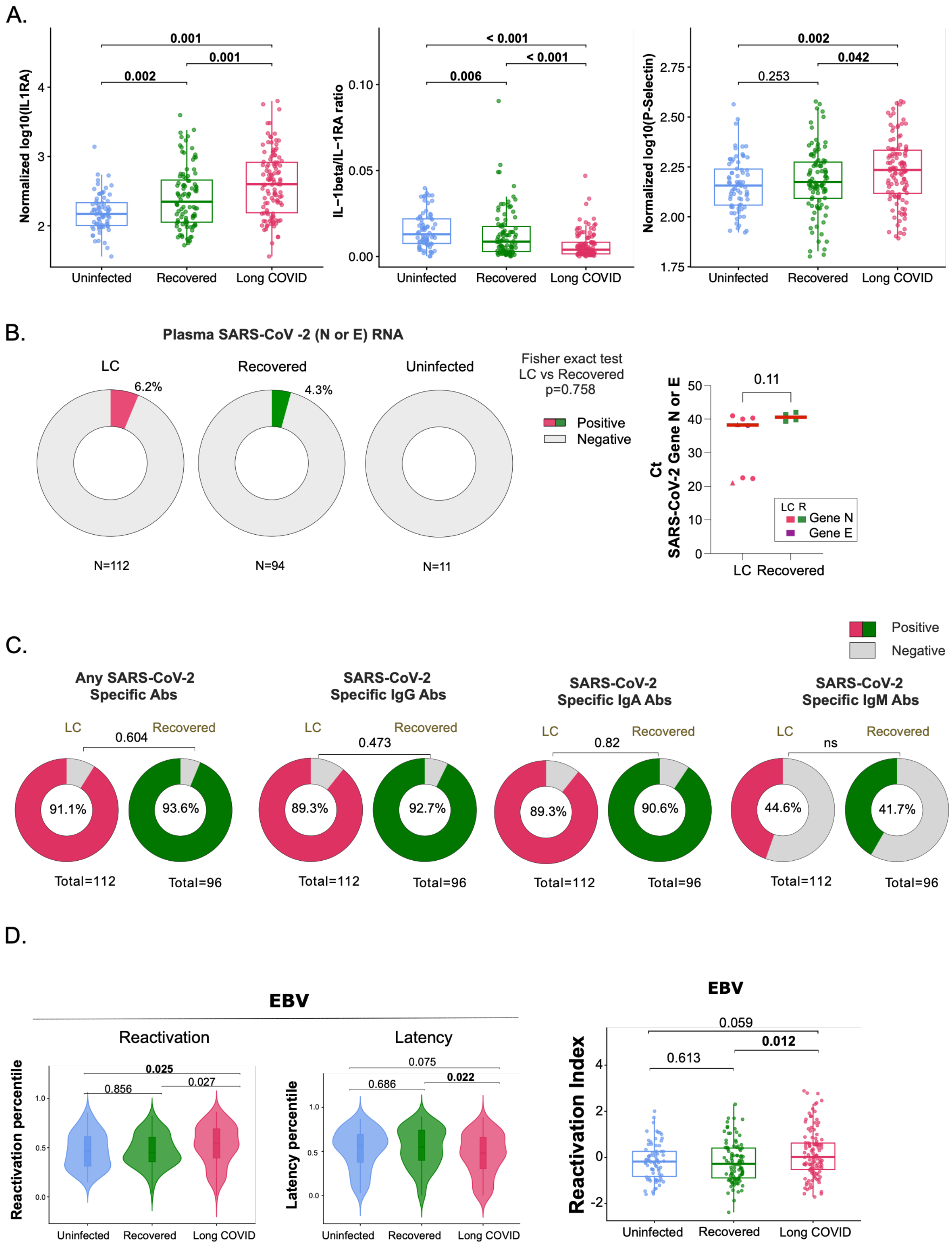

Supplementary Figure 6. Sensitivity analyses excluding PWLC whose initial SARS-CoV-2 infection was based solely on clinical diagnosis. All analyses were repeated after excluding PWLC without laboratory confirmation of the initial SARS-CoV-2 infection. (A) Comparison of normalized log10-transformed IL-1RA levels, IL-1β/IL-1RA ratio, and normalized log10-transformed P-selectin levels among uninfected, recovered, and LC participants. (B) Detection of plasma SARS-CoV-2 RNA

targeting the N and/or E genes in LC, recovered, and uninfected participants. Donut plots show the proportion of participants with detectable plasma SARS-CoV-2 RNA, and Ct values are shown for RNA-positive LC and recovered participants. (C) Proportion of LC and recovered participants with detectable SARS-CoV-2-specific antibodies, shown for any SARS-CoV-2-specific antibody and separately for IgG, IgA, and IgM. (D) EBV reactivation-associated and latency-associated antibody percentile scores and the EBV reactivation index across uninfected, recovered, and LC participants. The EBV reactivation index was calculated as the mean Z-score of reactivation-associated antibody responses minus the mean Z-score of latency-associated antibody responses. P-values for continuous variables were obtained using Kruskal-Wallis with Dunn tests with Holm correction, whereas differences in proportions were assessed using Fisher's exact test.

Supplementary Figure 7

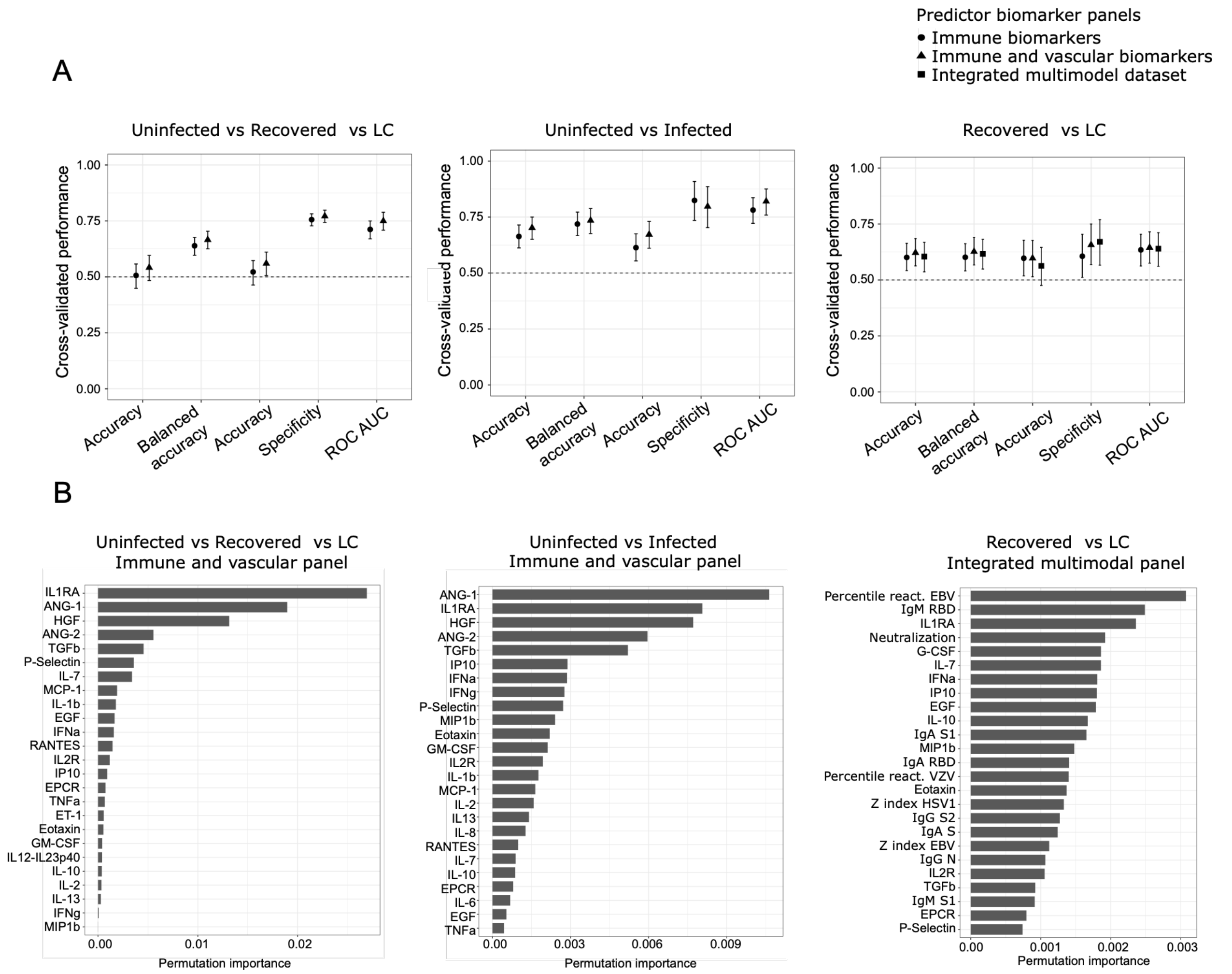

Supplementary Figure 7. Random forest classification performance and predictor importance across predefined biomarker panels. Random forest models were used to explore discrimination between study groups using three predefined predictor panels: 1) immune biomarker panel, comprising soluble immune biomarkers measured by Luminex together with TGF-β; 2) immune and vascular biomarker panel, additionally including markers of vascular/endothelial biology; and 3) integrated multimodal biomarker panel, incorporating immune and vascular biomarkers together with plasma SARS-CoV-2 RNA, SARS-CoV-2-specific antibody responses, and herpesvirus serology. (**A**) Cross-validated classification performance for three analytical tasks: discrimination among Uninfected, Recovered, and Long COVID (LC) participants (N=312); discrimination between previously infected participants (Recovered + LC) and Uninfected controls (N=312); and discrimination between Recovered and LC participants (N=238). The immune and immune-and-vascular panels were evaluated in all three tasks, whereas the integrated multimodal panel was evaluated for Recovered versus LC discrimination in the subset with complete multimodal data. Performance metrics shown are accuracy, balanced accuracy, sensitivity, specificity, and ROC AUC. Estimates represent out-of-fold performance from repeated stratified five-fold cross-validation with 20 repeats, with 95% confidence

intervals obtained by participant-level bootstrap resampling (2,000 iterations). The dashed horizontal line indicates chance-level performance. (**B**) Permutation-based variable importance for the predictor panel selected for each classification task: the immune and vascular panel for Uninfected versus Recovered versus LC (lef panel) and for Uninfected versus Infected (middle panel), and the integrated multimodal panel for Recovered versus LC (right panel).

Supplementary Figure 8

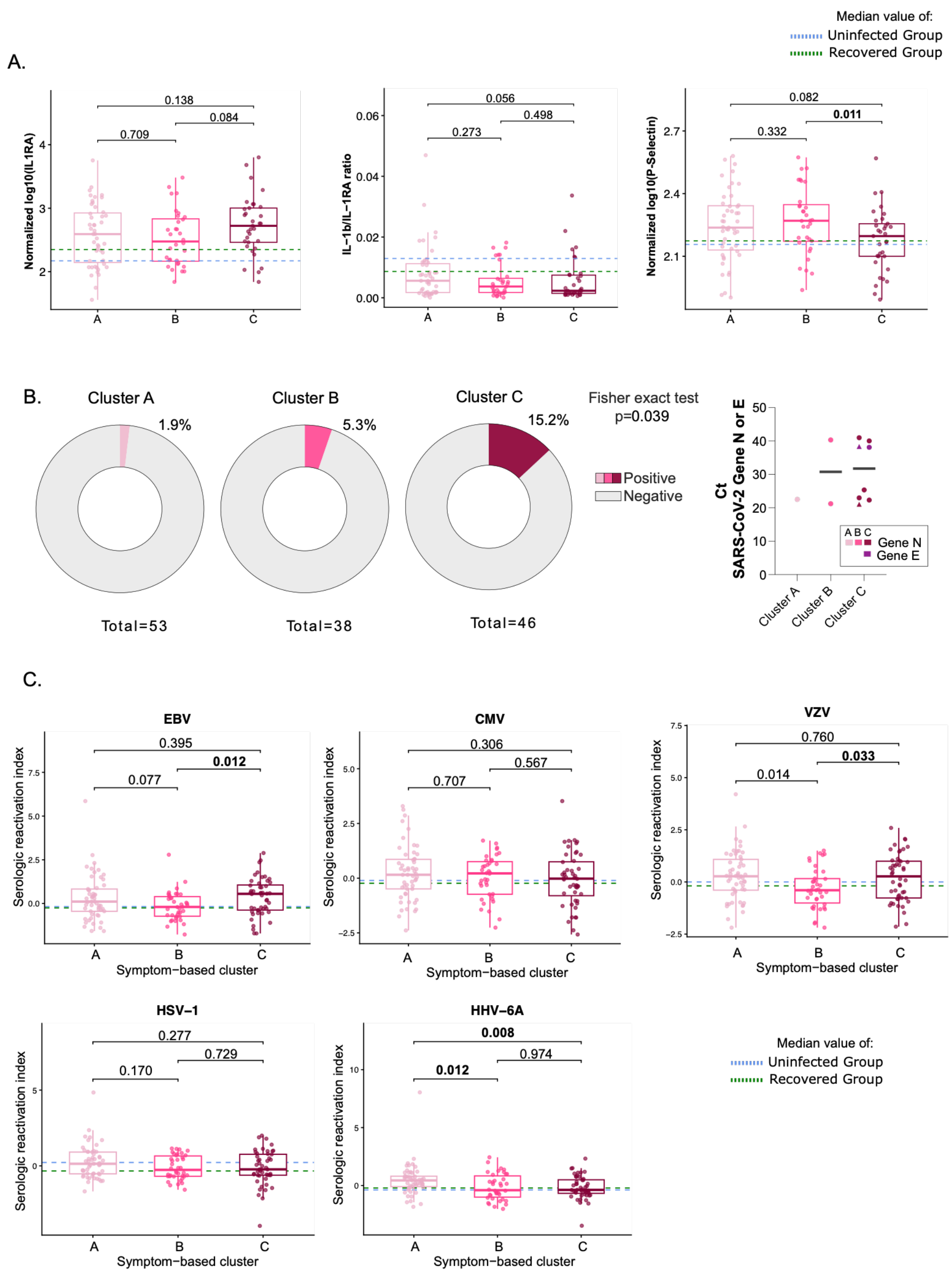

Supplementary Figure 8. Biological characteristics across symptom-based LC clusters. A) Normalized log10-transformed concentrations of IL-1RA (left), ratio IL1b/IL1RA (middle) and log10tranformed concentrations of P-selectin (right panel) across symptom-based LC clusters (A, B and C). **B**) Pie charts showing the proportion of participants with detectable plasma SARS-CoV-2 N/E RNA within each symptom-based cluster (A–C), together with scatter plots of cycle threshold (Ct) values among N RNA-positive individuals. **C**) Reactivation indices for EBV, VZV, CMV, HSV1, and HHV6A across symptom-based clusters. Blue and green dashed lines represent the median values of uninfected controls and recovered individuals, respectively. For A and C, statistical comparisons were performed using Kruskal–Wallis tests followed by Dunn's post-hoc tests with holm correction. Exact

adjusted p-values are indicated in the figure. Center lines represent medians, boxes indicate interquartile ranges, and whiskers represent 1.5× the interquartile range. The discontinuous horizontal lines represent the median of both Uninfected (blue) and Recovered (green) groups.
